# A screening strategy for identifying the developmental and reproductive toxicity potential of botanicals

**DOI:** 10.1080/13880209.2026.2659421

**Published:** 2026-04-28

**Authors:** Catherine Mahony, Arianna Bartlett, Suzanne Fitzpatrick, Corrado Galli, Piper Hunt, Amy Inselman, Jessica Jimenez, Julie Krzykwa, Jacob Larson, Cynthia Rider, John Rogers, Vicki Sutherland, Kevin Welch, Constance A. Mitchell

**Affiliations:** ^a^ Procter & Gamble Technical Centre, Reading, UK; ^b^ Research Institute for Fragrance Materials, Inc., Mahwah, NJ, USA; ^c^ US FDA, Human Foods Program, Office of Chemistry & Toxicology, Laurel, MD, USA; ^d^ Section of Toxicology and Risk Assessment, Department of Pharmacological and Biomolecular Sciences (DISFeB), University of Milan, Milan, Italy; ^e^ US FDA, National Center of Toxicological Research, Jefferson, AR, USA; ^f^ Procter & Gamble Healthcare, Cincinnati, OH, USA; ^g^ Health and Environmental Sciences Institute, Washington, DC, USA; ^h^ Herbalife International of America Inc, Torrance, CA, USA; ^i^ Division of Translational Toxicology, National Institute of Environmental Health Sciences, Durham, NC, USA; ^j^ ToxStrategies LLC, Chapel Hill, NC, USA; ^k^ Johnson & Johnson Medtech, Brunswick, NJ, USA; ^l^ USDA-ARS, Poisonous Plant Research Laboratory, Logan, UT, USA

**Keywords:** Botanicals, developmental, natural products, NAMs, reproduction, supplements

## Abstract

**Context:**

Botanicals are widely consumed as dietary supplements and traditional medicines, yet their developmental and reproductive toxicity (DART) potential is often inadequately characterized. Botanicals are chemically complex and variable, complicating traditional *in vivo* testing and interpretation. There is a need for fit-for-purpose new approach methodologies (NAMs) to evaluate DART hazards of complex mixtures while reducing reliance on mammalian models.

**Objective:**

This review describes the Botanical Safety Consortium (BSC) DART Working Group strategy to evaluate a battery of NAMs for screening botanical extracts and outlines the botanicals selected as case studies to assess assay suitability for complex mixtures.

**Methods:**

Available NAMs relevant to DART were identified and reviewed, including human induced pluripotent stem cell assays (e.g., devTox Quick Predict), zebrafish embryos, *Caenorhabditis elegans*, transcriptomics with Connectivity Map analysis, *in vitro* pharmacology profiling, and selected *in silico* tools. Assays were selected to provide complementary functional, mechanistic, and biomarker coverage across biological levels. Eighteen botanicals were chosen based on published *in vivo*, mechanistic, livestock, or *in vitro* evidence indicating no concern, uncertain effects, or established DART hazards.

**Results:**

The selected NAM battery captures diverse mechanisms relevant to DART. The case study botanicals span clear positives (e.g., locoweed, poison hemlock, cottonseed), suspected positives based on limited data (e.g., bitter melon, goldenseal, rue), and low concern examples (e.g., Asian ginseng), enabling evaluation of assay performance for complex mixtures.

**Conclusions:**

This strategy paper lays the groundwork to assess the suitability of integrated NAMs for screening botanical DART potential and establishes the foundation for subsequent testing and case study evaluation.

## Introduction

Botanicals used in herbal supplements and traditional medicines are consumed by a large portion of the global population (Paine and Roe 2018). In the United States, retail sales of herbal supplements have increased annually since 2004, reaching a reported $12.6 billion in 2023 (Smith et al. 2024). Although the term ‘botanical’ primarily refers to plants, it also includes algae and fungi. These materials are chemically complex, often containing hundreds to thousands of compounds. The composition of finished extracts can vary depending on factors such as growing conditions, extraction methods, co-formulation with other botanicals, and potential adulteration (Mitchell C et al. 2022). This variability complicates toxicological evaluation, especially in comparison to single-ingredient substances like pharmaceuticals or agrochemicals.

Developmental and reproductive toxicity (DART) is a critical endpoint for botanical safety assessment. While some regulatory frameworks require toxicity testing for botanicals (Low et al. 2017), historical use often serves as the primary basis for evaluating risk. In certain regions, regulatory intervention occurs only after adverse outcomes are reported post-marketing. This reactive model emphasizes the need for proactive tools that can rapidly screen for DART potential. Traditional *in vivo* testing is resource-intensive and complicated by the natural variability of botanical mixtures. Selecting a single, representative test material is difficult, and testing multiple samples is rarely practical (Mitchell et al. 2022). This creates a clear need for alternative predictive assays capable of evaluating DART hazards in complex botanical mixtures. There is growing momentum to reduce reliance on vertebrate animal models for toxicity testing (Bos et al. 2020; FDA 2024), and a variety of non-vertebrate and *in vitro* models have been developed to replace, reduce, or refine traditional mammalian studies, including those that address DART endpoints. These assays can offer valuable insight into developmental hazards, but most have not yet been broadly applied to botanicals.

To address this gap, the current effort evaluates several new approach methodologies (NAMs) for screening botanical mixtures for DART potential. This manuscript describes the existing NAMs being utilized and the well-characterized botanicals with existing DART-relevant data that will be used to assess the assays. This work is being conducted within the Botanical Safety Consortium (BSC; botanicalsafetyconsortium.org), a global, cross-sector initiative dedicated to advancing botanical safety through the application of modern toxicological tools. While the present study focuses on specific assays and materials, the BSC framework supports continued evaluation of additional methodologies to assess other endpoints such as hepatotoxicity, cardiotoxicity, neurotoxicity, genotoxicity, and dermal toxicity (Mitchell et al. 2022; Mitchell et al. 2025).

## The importance of testing for DART in characterizing the safety of botanicals

DART refers to adverse effects on sexual function, fertility, pregnancy outcomes, fetal development, or postnatal growth and maturation resulting from exposure to a substance. DART testing is essential for identifying risks to reproductive health, including the potential for birth defects, impaired fertility, and functional abnormalities that may not emerge until later in life.

Several standardized *in vivo* assays are widely used to assess DART potential, particularly in rodents. These include the OECD Test Guidelines 414 (Prenatal Developmental Toxicity Study), 421 (Reproductive/Developmental Toxicity Screening Test), and 443 (Extended One-Generation Reproductive Toxicity Study). These models have been instrumental in identifying chemical hazards during sensitive windows of development and reproduction. However, ethical considerations, regulatory shifts, and the high resource demands of traditional DART studies underscore the importance of NAMs tailored to complex substances such as botanicals.

**Figure 1.**
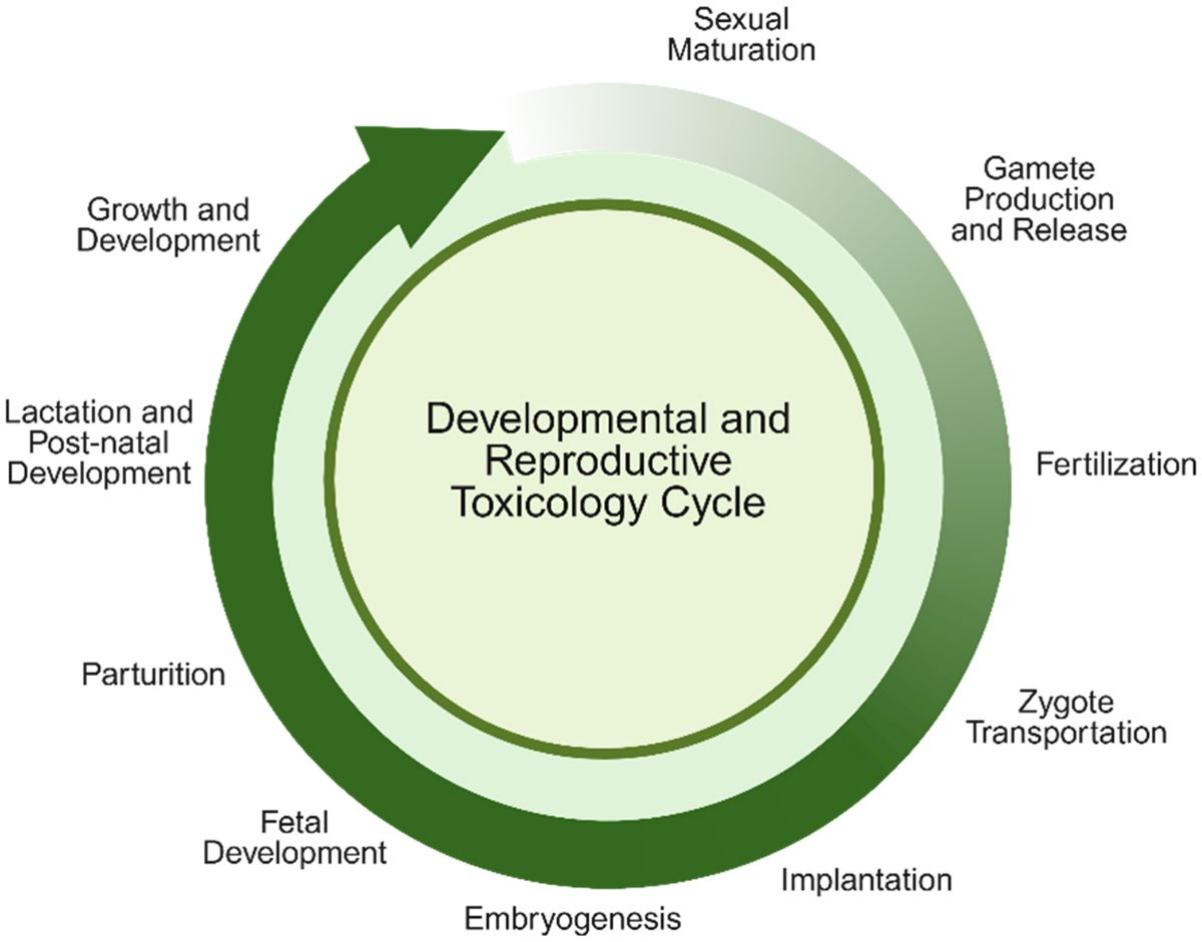
Overview of the developmental and reproductive cycle. Adapted from Foster and Gray (2019) and Hannas et al. (2025). Figure made with BioRender.

Adverse outcomes during development or reproduction may result from exposures that are not evident through human history-of-use alone, especially given the delayed and sometimes subtle nature of developmental effects. These can include birth defects, reduced growth, or disruptions in physiological function that may not become apparent until adulthood (Figure 1). Consequently, robust DART testing plays a critical role in identifying risks to pregnant individuals, developing fetuses, and reproductive health more broadly.

Historically, DART endpoints have been assessed in rodent or rabbit models. However, published botanical DART studies remain limited, and the available literature often suffers from issues that reduce interpretability. Common problems include failure to adhere to standardized test guidelines or good laboratory practices (GLP), lack of detailed reporting, and poor characterization of the test material. Even when high-quality data are available, differences in botanical composition across preparations can make it difficult to extrapolate findings. These limitations further highlight the need for additional, well-controlled research using NAM approaches to evaluate DART risks in botanical mixtures.

## NAMs for DART evaluation

Considering the challenges described above, the BSC DART Working Group has focused on identifying NAMs to support screening and prioritization of botanicals for DART potential. NAMs encompass a variety of non-animal and alternative model systems, ranging from invertebrate organisms to advanced *in vitro* platforms, capable of providing mechanistic, functional, and biomarker-based insights. Importantly, the use of NAMs aligns with the 3Rs principles (reduce, refine, replace) by minimizing reliance on mammalian testing while enhancing mechanistic understanding of toxicity pathways relevant to development and reproduction.

Given that DART outcomes can arise from perturbations to diverse biological processes, no single assay can capture the full spectrum of potential effects. As such, a battery approach is necessary, employing complementary assays that together address multiple aspects of developmental and reproductive biology. The battery under evaluation includes model organisms such as zebrafish (*Danio rerio*) embryos and the nematode *Caenorhabditis elegans*, both of which offer the advantages of whole-organism biology within non-protected life stages. These models are supplemented by *in vitro* stem cell-based assays that evaluate early developmental processes, transcriptomic analyses that provide mechanistic insights at the gene expression level, and safety pharmacology screens that assess critical functional targets. Together, these approaches are anticipated to provide a framework for evaluating the DART potential of botanicals in a scientifically rigorous and animal-reducing manner.

**Figure 2.**
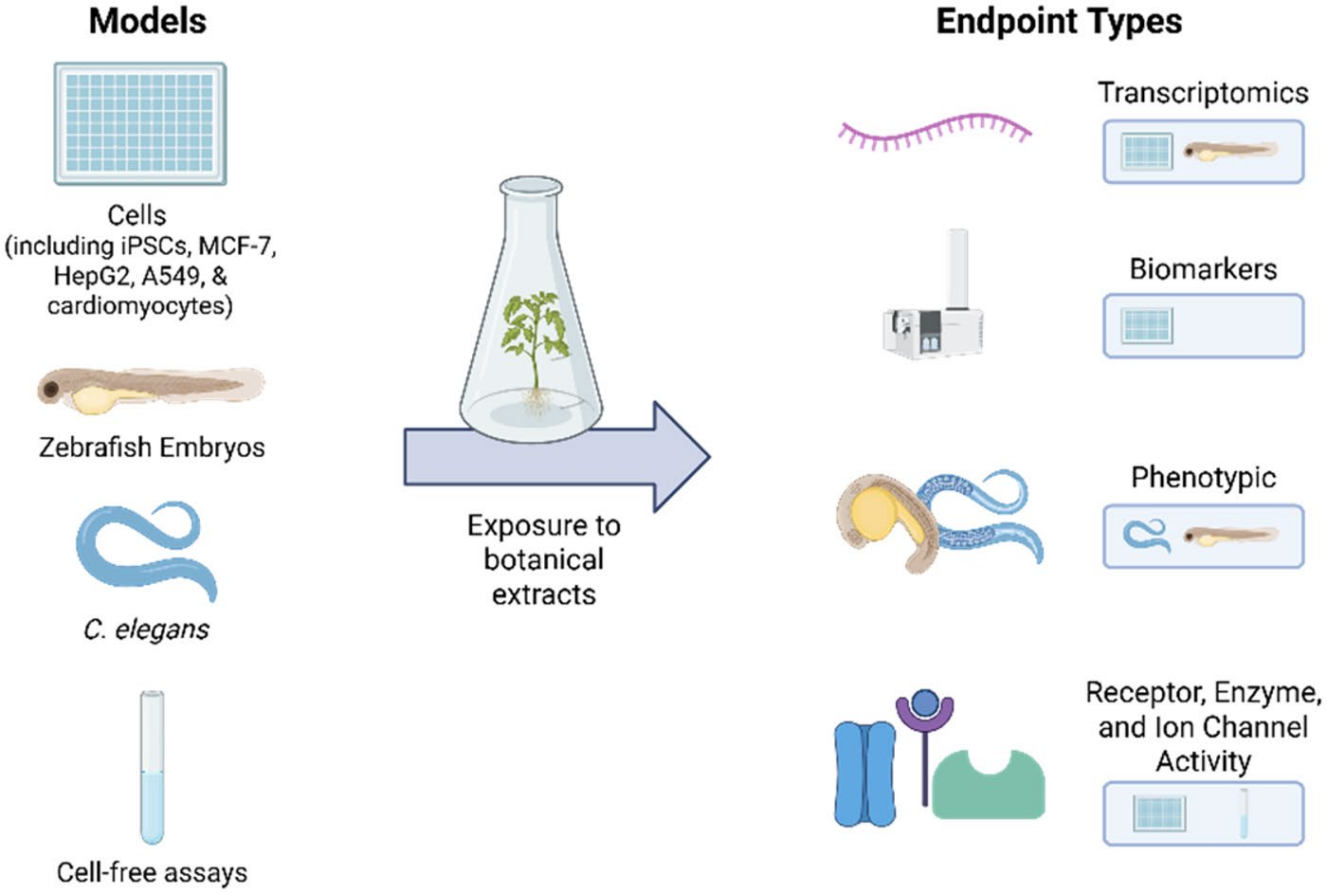
Overview of the model systems and endpoint types used to determine the suitability of these assays for botanicals. Figure created with BioRender.

In the following section, we briefly describe each assay, outlining its biological model, purpose, relevance to DART assessment, and primary endpoints. Figure 2 shows an overview of the selected models and endpoint types. We summarize key methodological features, including assay design, execution, and any relevant validation efforts. Where available, we highlight applications of botanicals to illustrate their role in natural product safety evaluation.

## *In vitro* stem cell assays for biomarker investigation

Human embryonic stem cells (hESCs) and human induced pluripotent stem cells (iPSCs) are utilized to predict developmental toxicity. Like hESCs, iPSCs are reprogrammed cells capable of differentiating into any cell type, but do not originate from embryos. In our investigations, we will concentrate on an assay employing iPSCs.

**Figure 3.**
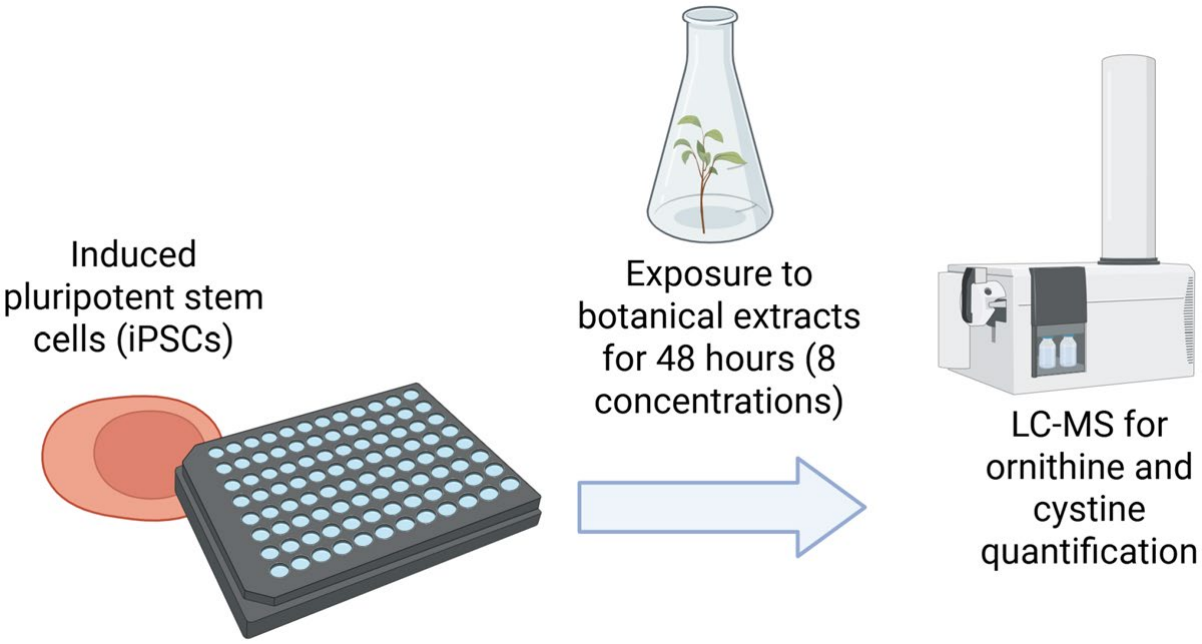
Schematic diagram illustrating the devTox Quick Predict assay. Human-induced pluripotent stem cells (iPSCs), reprogrammed to differentiate into any cell type without the use of embryonic material, are plated onto well plates and exposed to botanical extracts across eight different concentrations for a 48-h period. Following exposure, cells are analyzed using liquid chromatography-mass spectrometry (LC-MS) to quantify the ratio of ornithine to cystine, two metabolites empirically identified as predictive biomarkers of developmental toxicity. Created with BioRender.

One assay, the devTox Quick Predict assay (Palmer et al. 2013), evaluates the ratio between ornithine and cystine following a two-day exposure of iPSCs to eight different concentrations of a test substance (Figure 3). This method measures cell viability to assess potential teratogenic responses induced either directly by (in our case) the botanical itself or by cytotoxicity. The use of a concentration range enables comparison of bioactive responses to levels that may be relevant to expected human exposure, supporting interpretation of assay results in a dose-contextualized manner.

Ornithine and cystine were selected based on metabolic profiling of hESCs exposed to 23 pharmaceutical compounds known to cause developmental toxicity, including cardiovascular, skeletal, and craniofacial defects (Palmer et al. 2013). Ornithine supports nitrogen balance and polyamine synthesis, both essential for cell growth and differentiation (Pegg 2009). Cystine, converted to cysteine upon cellular uptake, contributes to glutathione production and redox regulation, which are critical for protecting against oxidative stress during development (Hansen 2006; Palmer et al. 2013).

Zurlinden et al. evaluated the devTox Quick Predict assay using over 1,000 chemicals from the ToxCast library (Zurlinden et al. 2020). The assay demonstrated strong specificity (>84%) and overall accuracy (79–82%) in identifying developmental toxicants, though sensitivity was somewhat lower (<67%). These findings suggest the assay performs well in correctly identifying non-toxicants, with room for improvement in detecting certain developmental toxicants, especially those with mechanisms not captured by the current test system or those with lower potency. The authors also noted the absence of metabolic competence as a consideration when interpreting results from this *in vitro* model.

The Reprotracker assay, like the devTox Quick Predict, uses iPSCs differentiated into cardiomyocytes, hepatocytes, and neural rosettes in the presence of test compounds (Jamalpoor et al. 2022). It assesses morphology, cardiomyocyte function, and biomarker gene expression to monitor lineage-specific differentiation. Key markers include BMP and MYH6 (mesoderm/cardiomyocytes), FOXA2 and AFP (endoderm/hepatocytes), PAX6 and Nestin (ectoderm/neural rosettes), and OCT4 (pluripotency). Validation with 33 compounds showed 85% sensitivity and 84% specificity (Toxys 2023).

To our knowledge, these tools have not been explored with botanicals as complex mixtures.

## Alternative animal models

The use of multiple alternative models, including *in silico* tools, *in vitro* assays, and non-mammalian small organisms, within tiered or battery testing approaches is widely encouraged by regulatory guidelines and legislative frameworks (ECHA, 2015; FDA 2024). Current cell-based systems alone cannot model all aspects of *in vivo* DART functions, creating potential data gaps (Becker et al. 2022). Conservation of key genetic pathways regulating organismal development, as well as high levels of concordance for adverse chemical effects on development, supports the use of apical endpoint testing in non-mammalian small model organisms such as zebrafish and *C. elegans* to fill these gaps (Leung et al. 2008; Padilla et al. 2012; Boyd et al. 2016; Masjosthusmann et al. 2018).

Zebrafish embryos and larvae (before independent feeding) and *Caenorhabditis elegans* at all life stages are not considered animals under most regulatory frameworks, enabling ethically streamlined testing (Racz et al. 2017). Both species offer practical advantages for toxicology: their transparency allows for real-time visualization of organ development and pathology in intact organisms; their small size and compatibility with liquid media support high-throughput screening of individual chemicals or mixtures across a broad range of concentrations at significantly reduced cost compared to mammalian models (Padilla et al. 2012; Boyd et al. 2016). Zebrafish and *C. elegans* also share strong genetic conservation with humans, with over 70% of human genes represented by homologs in each species—and nearly 90% for known human disease genes, supporting their relevance for human health hazard evaluation (Kuwabara and O’Neil 2001; Howe et al. 2013). Exposure routes differ (i.e., zebrafish primarily absorb chemicals across embryonic membranes, while *C. elegans* exposures are largely oral) making them complementary tools for toxicity evaluation. Both models support the assessment of key apical endpoints, such as growth and locomotion, which are predictive of developmental toxicity in mammals (Hamm et al. 2024). These endpoints offer mechanism-agnostic insight into chemically induced perturbations of complex biological processes and have been proposed as part of new approach methodologies for developmental and neurotoxicity testing (Masjosthusmann et al. 2018).

More details on each model are provided below. While these two models offer advantages in capturing DART responses across multiple levels of organization from molecular to organismal and reflect better the complex processes of embryonic development *versus* single cell systems, they are not without their limitations (van der Ven 2010). There are differences in receptor isoforms, expression levels, and tissue distribution, which likely affect sensitivity and specificity to toxicants and complicate human relevance (Gamse and Gorelick 2016). Notably, using *C. elegans* or zebrafish embryos to predict developmental toxicity of around 1,000 chemicals in the ToxCast library yielded a balanced sensitivity and specificity ranging from ∼45 to 53%, which is modest, but still only slightly lower than the 58% balanced predictivity of rat and rabbit for each other (Boyd et al. 2016). Importantly for screening, the positive predictivity of both models is in the 70% range, while the true negative predictivity is much lower (Boyd et al. 2016), indicating higher confidence for hazard identification, but lower confidence in determining human safe levels. Botanicals have been explored with zebrafish embryos (Chahardehi et al. 2020) and *C. elegans* (Wang et al. 2024) in a few cases, but there has not been an evaluation of their performance with botanicals and the suitability has not been done to our knowledge.

Overall, these alternative models are proposed here primarily as part of a screening and prioritization strategy for botanical DART potential.

### Caenorhabditis elegans *for functional investigation*

*Caenorhabditis elegans* are small, nonpathogenic nematodes with an adult length of ∼1 mm. *C. elegans* was the first multicellular eukaryote with a fully sequenced genome and has been central to discoveries in conserved cellular biology (Consortium 1998). They have a tough external cuticle and a metabolically active digestive system with mammalian-analogous components (Hunt 2017). In laboratory settings, the *C. elegans* three-day life cycle and ∼3-week lifespan allow for rapid acute and chronic toxicity testing at different life stages critical to development and reproduction. *C. elegans* have a distinct metabolic profile compared to mammals, with differences in xenobiotic metabolism pathways that may influence chemical bioavailability and activity (Harlow et al. 2018). While useful for detecting organism-level effects, results should be interpreted with caution for compounds requiring specific metabolic activation.

Key *C. elegans* DART-related endpoints include growth rates, reproductive output, and behavioral changes that can indicate neurotoxicity. Development can be quantified by measuring worm body size or reproductive tract maturity at specified intervals. Reproductive toxicity is a difficult endpoint to replace with alternative assays because so many different systems are involved. While *C. elegans* do not form a placenta or have cyclical fertility periods, reproductive functions that are conserved from worms to humans include many aspects of oocyte maturation, oocyte quality control, and epigenetic genome remodeling, as well as genetic regulation of embryonic tissue patterning *via* early cell divisions and polarity processes (Athar and Templeman 2022).

**Figure 4.**
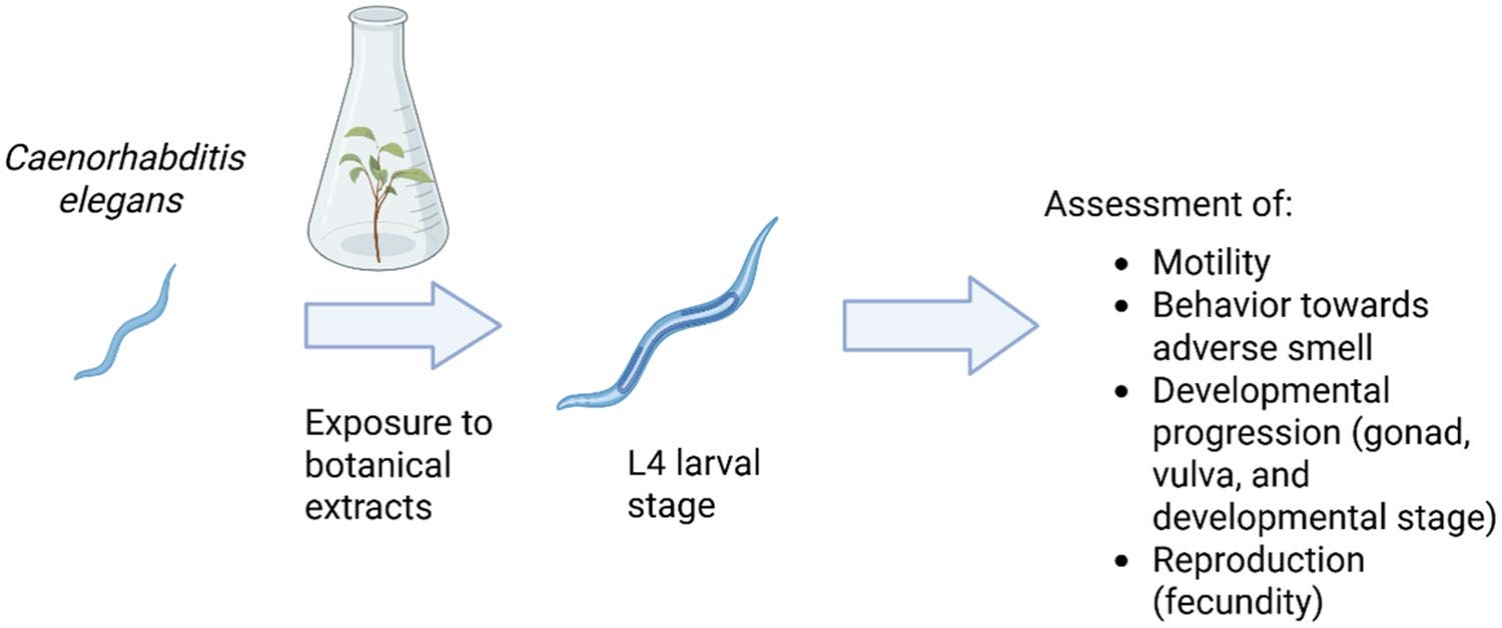
Schematic diagram illustrating the use of *C. elegans* to screen for general, developmental, and reproductive toxicity. L4: 4th larval stage. Figure created with BioRender.

Though there are various experimental set-ups that can be used for *C. elegans*, we are exploring the use of exposing adults and following the eggs laid through the L4 phase (young adult) (Figure 4). Several endpoints can be measured, including general developmental, gonad, vulva, developmental stage, and fecundity. The BSC is also using this model organism for assessing neurotoxicity, including assessing motility and behavior (Kanungo et al. 2024).

### Zebrafish embryos for functional investigation

Zebrafish are small fish widely used in embryology studies. Their high egg yield (about 200 per week) and external fertilization enable high-throughput testing. Zebrafish have a life cycle of three months and a lifespan of ∼3 years. Zebrafish embryos develop externally, facilitating toxicity testing without confounding maternal effects. All major zebrafish body systems are formed by 72 h post-fertilization (hpf), allowing tracking of developmental effects over time (Kimmel et al. 1995). In contrast to the simple *C. elegans* body plan, zebrafish have many organs in common with mammals, including a skeleton, eyes, a liver, and a circulatory system, all of which make zebrafish useful for studying xenobiotic effects on vertebrate-specific functions related to growth, neurogenesis, and morphogenesis (Adhish and Manjubala 2023). Zebrafish embryos possess some metabolic capability, including phase I and phase II enzymes, although this is developmentally regulated and may not fully recapitulate mammalian metabolism (Loerracher et al. 2020; Loerracher and Braunbeck 2021). Consequently, both parent compounds and early metabolites may contribute to observed effects, but species differences should be considered when interpreting results for human relevance.

**Figure 5.**
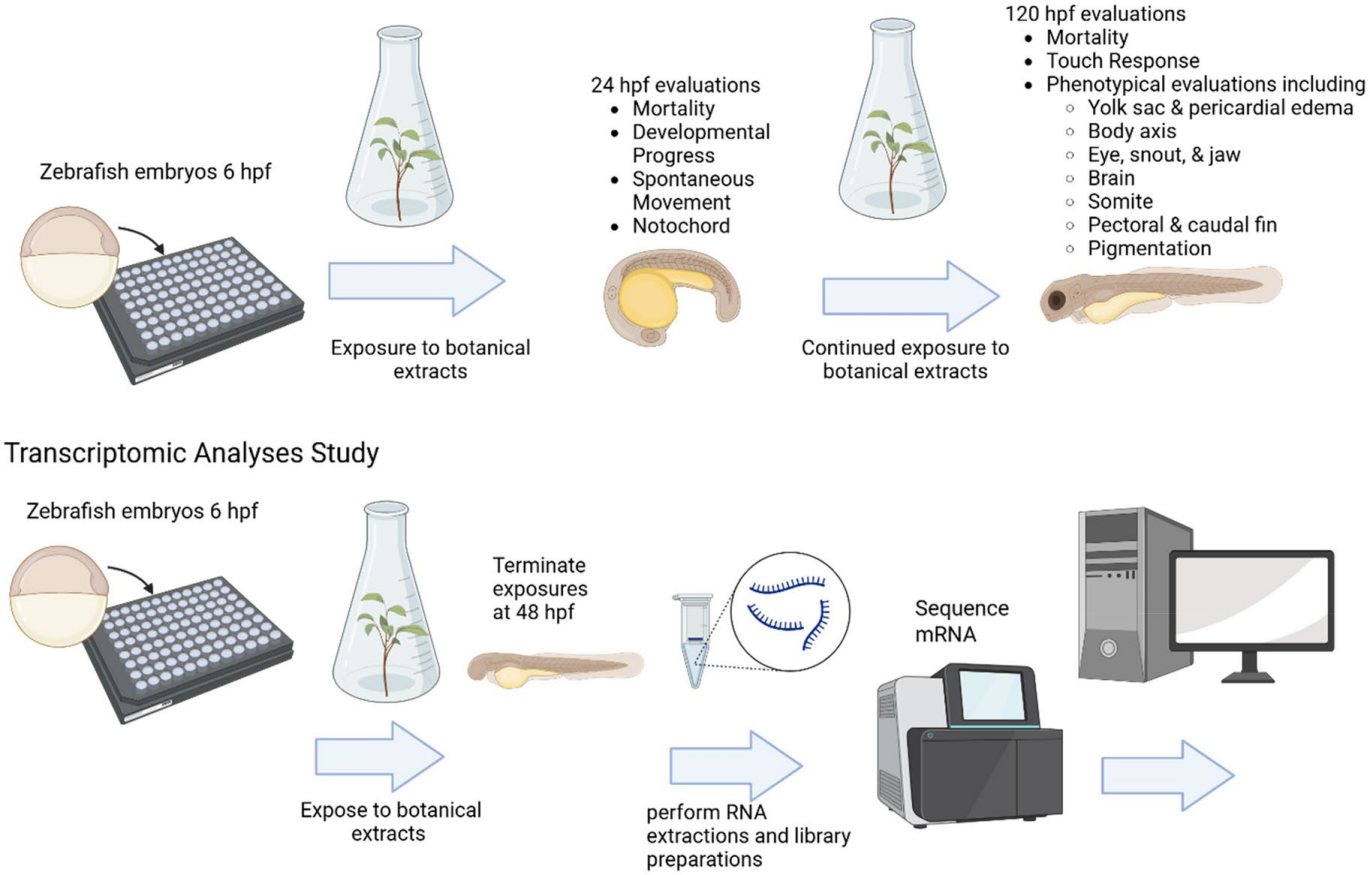
Schematic diagram illustrating the zebrafish embryo assay. Zebrafish embryos are dechorionated to enhance chemical exposure and assessed at 24 and 120 h post-fertilization (hpf) for mortality, developmental progression, spontaneous movement, notochord morphology, and photomotor responses. At 120 hpf, additional neurodevelopmental and morphological endpoints are evaluated. Transcriptomic analysis at 48 hpf is used to explore potency and mechanistic pathways. Figure created with BioRender.

Similar to *C. elegans,* there are many experimental setups for zebrafish. Here we describe one common approach. Zebrafish embryos are exposed to test substances *via* absorption from the medium, with enzymatic dechorionation enhancing xenobiotic bioavailability (Truong and Tanguay 2017). Mortality, a measure of systemic toxicity akin to a cytotoxicity assay, is assessed at 24 and 120 hpf (Figure 5). Developmental endpoints at 24 hpf include progression, spontaneous movement, notochord morphology, and photomotor response, which measures embryonic muscle innervation by response to light stimulation (Reif et al. 2016). At 120 hpf, endpoints assessed include the larval photomotor response, acoustic startle response, and comprehensive morphological evaluation to detect teratogenesis and organ-specific effects (Truong et al. 2011).

Mechanistic insights can also be gained using omics approaches (e.g., transcriptomics), morpholino-mediated gene knockdowns, or immunohistochemical staining. For the BSC, we plan to leverage transcriptomic analyses at 48 hpf to inform potency and mechanistic interpretation.

## Transcriptomics and connectivity mapping for mechanistic information

Since the early 2000s, quantification of mRNA levels in cells or organisms has been used in toxicology to understand molecular perturbations. Gene expression can be measured in multiple ways, from microarrays to whole transcriptome sequencing or targeted gene panels. There have been efforts to ensure quality and gene-wise test statistics are used for differential expression analysis and to assemble gene signatures for test substances (Harrill et al. 2021).

**Figure 6.**
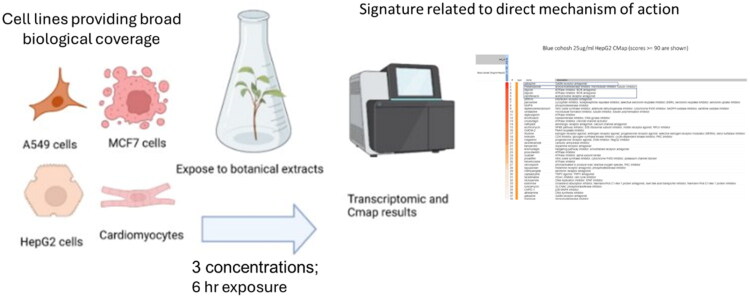
Schematic diagram illustrating the transcriptomics assay and Connectivity Map (CMap) analysis. Four cell lines: MCF-7, A549, HepG2, and iCell cardiomyocytes are utilized to ensure broad biological coverage and test concentrations are selected to be non-cytotoxic. Following a 6-h exposure, to ensure transcriptional signatures reflect direct mechanisms of action, transcriptomic data are collected and analyzed using CMap, which compares gene expression signatures to a large reference database to infer functional similarities. Figure created with BioRender.

While many organisms or cell types could be used, here we describe previously published methods to obtain mechanistic information across multiple cell types. Four cell types are to be used in our transcriptomic experiments (Figure 6): MCF-7 (breast epithelial adenocarcinoma), A549 (lung epithelial carcinoma), HepG2 (hepatocellular carcinoma), and iCell cardiomyocytes (derived from iPSCs). Previous work has shown that these four cell types respond to a diverse range of chemicals as well as botanical extracts and allows for a broad ‘biological coverage’ (VanderMolen et al. 2020). Test concentrations must be non-cytotoxic to obtain meaningful data (e.g., <20% by a cell viability assay) to obtain a gene signature related to a direct mechanism of action (Lamb et al. 2006; De Abrew et al. 2019).

Tools like the Connectivity Map (CMap) further enhance mechanistic interpretation by comparing compound-induced transcriptional signatures to a large reference database of gene expression profiles (Lamb et al. 2006). These comparisons can suggest shared mechanisms, pathway perturbations, or functional similarities between tested compounds and known reference agents. Recent refinements to the CMap, including expanded datasets and scoring algorithms such as the Characteristic Direction method, have improved its utility for screening and mode-of-action inference (Subramanian et al. 2017).

By capturing whole genome transcriptional responses in multiple cell types, it is possible to cast a wide net around diverse and potentially novel modes of action, consequently enhancing mechanistic understanding. However, there are limitations to using immortalized cell lines or other monolayer cell cultures as they do not fully recapitulate the complexity of a biological system. *In vitro* transcriptomic assays may have limited metabolic capacity, depending on the cell types used, which could affect detection of pathways triggered by metabolites rather than parent compounds. However, these approaches can still provide insight into xenobiotic response pathways (e.g., AhR, PXR) and downstream effects. Use of metabolically competent systems or integration with metabolic predictions can improve interpretation.

Transcriptomic data can also be used to derive benchmark concentrations (BMCs) or transcriptomic points of departure (tPODs), which enable potency comparisons across chemicals. These approaches, from short term *in vivo* studies and other models like zebrafish embryos, estimate the concentration at which coordinated transcriptional changes associated with potential adverse effects are not expected to occur (O’Brien et al. 2025). Analogous to benchmark doses used in traditional toxicology, tPODs and BMCs require dose-response data from at least three test concentrations, though higher resolution with five or more concentrations is preferred for greater confidence.

## Pharmacology screen for mechanistic information

*In vitro* pharmacological profiling is routinely used by the pharmaceutical industry and more recently has been proposed by the cosmetic industry (Burbank et al. 2024). It includes enzymes, receptors, ion channels, and transporters whose involvement is either known or suspected to induce adverse effects in humans. Previous work has shown that these targets respond as well to botanical extracts (VanderMolen et al. 2020). We are exploring a panel of 83 assays offerred by Eurofins as described by Burbank et al. (2024), including assays to assess binding at various targets associated with DART (Table 1) (e.g., estrogen, androgen, retinoic acid, progesterone, and thyroid hormone receptors, as well activity toward ion channels and angiotensin converting enzymes, and select kinases) (Wu et al. 2013; Burbank et al. 2024).

**Table 1.** A list of DART relevant assays included in the pharmacology profiling panel.

Target	Target	Target	Target	Target
Alpha adrenergic	Ion channels	Beta adrenergic	Angiotensin converting enzymes	Opioid
Assay	Assay	Assay	Assay	Assay
alpha2B Human Adrenoceptor GPCR Binding Antagonist Radioligand LeadHunter Assay	nAChR (alpha4/beta2) Human Ion Channel Binding Agonist Radioligand LeadHunter Assay hERG Human Potassium Ion Channel [3H] Dofetilide Binding Antagonist Radioligand SafetyScreen Sodium Channel Site2 (Nonselective) Rat Ion Channel Batrachotoxin Mass Spectrometry Binding LeadHunter Assay	beta1 Human Adrenoceptor GPCR Binding Agonist Radioligand LeadHunter Assay beta2 Human Adrenoceptor GPCR Binding Antagonist Radioligand LeadHunter Assay	ACE Human Angiotensin- Converting Metallo Peptidase Enzymatic LeadHunter Assay ACE2 Human Angiotensin- Converting Metallo Peptidase Enzymatic LeadHunter Assay	mu (MOP) Human Opioid GPCR Binding Agonist Radioligand LeadHunter Assay
Target	Target	Target	Target	Target
Aromatase	Angiotensin	Cyclooxygenases	Estrogen	Acetylcholinesterase
Assay	Assay	Assay	Assay	Assay
CYP19A1 Human CYP450 Enzymatic LeadHunter Assay	AT1 Human Angiotensin GPCR Binding Antagonist Radioligand LeadHunter Assay	COX1 Human Cyclooxygenase Enzymatic LeadHunter Assay COX2 Human Cyclooxygenase Enzymatic LeadHunter Assay	ERalpha Human Estrogen NHR Binding Agonist Radioligand LeadHunter Assay ERbeta Human Estrogen NHR Binding Agonist Radioligand LeadHunter Assay	Acetylcholinesterase Human Enzymatic LeadHunter Assay
Target	Target	Target	Target	Target
Androgen	HMG-CoA reductase	Serotonin	Kinases	PPAR
Assay	Assay	Assay	Assay	Assay
AR Human Androgen NHR Binding Agonist Radioligand LeadHunter Assay	HMG-CoA Reductase Human Enzymatic LeadHunter Assay	5-HT2B Human Serotonin GPCR 125IDOI Binding Agonist Radioligand LeadHunter Assay	KDR (VEGFR2) (KDR) Human RTK Kinase Enzymatic Radiometric [Km ATP] KinaseProfiler LeadHunter Assay FGFR3 Human RTK Kinase Enzymatic Radiometric [Km ATP] KinaseProfiler LeadHunter Assay	PPARalpha Human NHR Binding Agonist Radioligand LeadHunter Assay PPARgamma Human NHR Binding Agonist Radioligand LeadHunter Assay
Target	Target	Target	Target	Target
Retinoic acid	Progesterone	Prostaglandin	Aryl hydrocarbon	Thyroid hormone
Assay	Assay	Assay	Assay	Assay
RARalpha Human Retinoic Acid NHR Binding Agonist Radioligand LeadHunter Assay	PR Human Progesterone NHR Binding Agonist Radioligand LeadHunter Assay	TP (TXA2) Human Prostanoid GPCR Cell Based Agonist Calcium Flux LeadHunter Assay TP (TXA2) Human Prostanoid GPCR Cell Based Antagonist Calcium Flux LeadHunter Assay	AhR Nuclear Receptor Assay (human, agonist)	TRalpha Human Thyroid Hormone NHR Functional Coactivator LeadHunter Assay TRbeta Human Thyroid Hormone NHR Binding Agonist Radioligand LeadHunter Assay

Panel includes other GPCRs, enzymes, ion channels, transporters and kinases not shown here.

Given that this panel was originally designed to support systemic toxicity assessment, it may also be informative for other endpoints relevant to botanical safety. In addition, inclusion or interpretation of targets related to xenobiotic sensing pathways, such as the aryl hydrocarbon receptor (AhR) and pregnane X receptor (PXR), can provide insight into mechanisms of action and potential impacts on metabolism. These pathways regulate the expression of genes involved in biotransformation and clearance, and their activation can influence both internal exposure and downstream biological effects. Integration with transcriptomic data, where available, can further support identification of pathway perturbation through characteristic gene expression signatures, aiding mechanistic interpretation of responses to complex botanical mixtures.

Assays are carried out with an initial single screening concentration, unless stated otherwise, in duplicate, along with respective reference chemicals. Results showing an inhibition or stimulation higher than 50% are considered to represent significant effects (i.e., a ‘hit’) and can be further investigated in multi-concentration assays to obtain an IC50 as a measure of potency.

A limitation of receptor- and enzyme-based pharmacology assays is the lack of metabolic competence, meaning that parent compounds are evaluated without accounting for potential bioactivation or detoxification. This is particularly relevant for botanical mixtures, where metabolites may drive biological activity. As such, results should be interpreted as indicative of direct target interactions and complemented with approaches that capture metabolism where relevant.

Whilst pharmacology screening can be aligned to modes of action associated with known developmental toxicants, the panel typically assesses a limited set of targets and does not capture the full complexity of development or metabolic competency which may activate or detoxify. Moreover, it depends on the availability of specific targets. For example, substances that cause toxicity *via* interaction with the Vitamin D receptor are not covered by this panel due to the unavailability of the receptor binding assay.

## *In silico* tools

*In silico* models for DART prediction have evolved to include a range of platforms such as quantitative structure–activity relationship (QSAR) models, structural alerts, and machine learning-based tools (Table 2). Examples include (but are not limited to) Derek Nexus (Lhasa Limited) (Ponting et al. 2022; Myden et al. 2024), which uses expert-derived rule-based alerts for reproductive and developmental endpoints, and the OECD QSAR Toolbox, which offers profilers and read-across tools (Schultz et al. 2018). Additional resources like ToxCast/Tox21 (Richard et al. 2016) and the VEGA platform incorporate mechanistic screening data and freely available predictive models (Danieli et al. 2023). A more integrated framework for DART is the Procter & Gamble (P&G) decision tree (DT) for developmental and reproductive toxicity, which is based on a detailed review of the literature, grouping chemicals with DART data according to receptor binding or chemical domain (Wu et al. 2013). The tree has been automated with versions used internally at P&G as well as in the OECD Toolbox and will identify compounds with structural features that are consistent with chemicals with a known precedent for DART effects. This workflow is especially valuable in data-limited contexts and has informed use under certain conditions for chemical safety assessment (Blackburn et al. 2015). We have investigated the applicability of the DART DT to a large phytochemical space (100,000 phytochemicals) and found that 7% and 81% mapped to structures and scaffolds (i.e., structural backbones), respectively, that carry a DART alert. Only 10% of phytochemicals did not yield an alert and 2% were not covered by the tree (Mitchell et al. 2022). The application of such tools to botanicals would therefore seem to be rather constrained, as not only may they be highly over-predictive, but they also require well-defined structures - parameters that are frequently poorly understood or variable in botanical mixtures. While *in silico* tools remain useful for prioritizing testing and identifying potential hazards, they are not yet reliable as stand-alone predictors of DART potential for botanicals and must be complemented by empirical and mechanistic data. However, if constituent structures are known, these tools can be used to help understand potential mechanisms to investigate further.

**Table 2.** *In silico* tools or databases reviewed for DART potential.

Tool/resource	Type	Purpose in DART assessment	Description	Access
Derek Nexus (Lhasa Limited)	Rule-based expert system	Structural alerts for DART hazards	Uses expert-curated rules to identify chemical features associated with reproductive and developmental toxicity endpoints	https://www.lhasalimited.org/products/derek-nexus.htm
OECD QSAR Toolbox	QSAR/read-across platform	Hazard identification and read-across	Provides profilers, structural alerts, and grouping tools to support read-across and prediction of DART-related effects	https://qsartoolbox.org
ToxCast/Tox21	High-throughput screening database	Mechanistic and bioactivity data	Large-scale *in vitro* assay data across biological targets that can inform potential DART-relevant mechanisms	https://www.epa.gov/chemical-research/toxicity-forecasting
VEGA platform	QSAR modeling platform	Predictive toxicology models	Provides a suite of QSAR models for toxicity prediction with applicability domain assessment to increase confidence	https://www.vegahub.eu
P&G DART Decision Tree	Structural alert framework/workflow	Identification of DART-relevant chemical features	Groups chemicals based on receptor binding and structural domains associated with developmental and reproductive toxicity; implemented in OECD Toolbox and internal workflows	https://qsartoolbox.org

## Botanicals case studies

To evaluate the suitability of assays for detecting DART potential of botanicals, case studies were identified based on relevance, available data, and scientific interest. Selection focused on botanicals with reported associations to DART endpoints to varying degrees and those that serve as informative examples (i.e., no expected *vs.* expected risk) for assessing assay performance (Tables 3 and 4, also available as Supplemental File 2). Botanicals selected by other Working Groups were reviewed for available DART data which also resulted in botanicals with unknown DART potential; these are described in Supplemental File 1. The rationale for selecting specific botanicals is described below (and summarized in Table 4). Literature cited in this review was identified through targeted searches of publicly available databases (e.g., PubMed, Google Scholar) and authoritative reports, with emphasis on studies relevant to developmental and reproductive toxicity and the selected botanicals. This was not intended to be a systematic review.

**Table 3.** List of botanicals, including their standardized common and scientific names, and the part(s) of the plant used to derive the botanical extract.

Standardized common name	Scientific name	Plant part(s)
*Aristolochia fangchi*	*Aristolochia fangchi* Y.C. Wu ex L.D. Chou & S.M. Hwang	Root
**Ashwagandha**	***Withania somnifera* (L.) Dunal**	**Root**
**Asian Ginseng**	***Panax ginseng* C.A. Mey.**	**Root**
**Bitter Melon**	***Momordica charantia* L.**	**Seed and fruit**
**Blue Cohosh**	***Caulophyllum thalictroides* (L.) Michx.**	**Root and rhizome**
Comfrey	*Symphytum officinale* L.	Root
**Cottonseed**	***Gossypium* spp.**	**Seed**
Ephedra	*Ephedra sinica* Stapf	Aerial parts
**False Hellebore**	***Veratrum album*** L.**, *Veratrum californicum*** L.	Root and rhizome
**Goldenseal**	***Hydrastis canadensis* L.**	**Root and rhizome**
Green Tea	*Camellia sinensis* (L.) Kuntze	Leaf
Kava	*Piper methysticum* G.Forst.	Root and rhizome
Kratom	*Mitragyna speciosa* (Korth.) Havil.	Leaf
**Locoweed**	***Astragalus* spp.; *Oxytropis* spp.**	**Aerial parts**
**Milk Thistle**	***Silybum marianum* (L.) Gaertn.**	**Seed**
**Poison Hemlock**	***Conium maculatum* L.**	**Whole plant**
**Rue**	***Ruta graveolens* L.**	**Leaf**
**Tree Tobacco**	***Nicotiana glauca* Graham**	**Leaf**
***Usnea***	***Usnea* spp.**	**Whole lichen**

Botanicals selected by the DART Working Group based on their DART profiles are in bold.

**Table 4.** Summary of selected botanicals for developmental and reproductive toxicity (DART) evaluation.

Botanical (common name)	Evidence for DART?	Evidence or lack thereof of DART phenotype	Evidence type	Details	Key references
*Aristolochia fangchi*	Developmental toxicity (teratogenic potential)	Malformations and developmental neurotoxicity	Zebrafish embryo, mechanistic (genotoxicity)	Zebrafish studies show developmental nephrotoxicity, neurotoxicity, and malformations. Aristolochic acids are well-characterized genotoxicants associated with human carcinogenicity, providing mechanistic support for developmental hazard.	Nortier and Vanherweghem 2002; Debelle et al. 2008; Phillips and Arlt 2009; Ding and Chen 2012; Chen et al. 2021
Ashwagandha	Uncertain DART potential	Mixed effects on fertility and hormonal endpoints; no evidence of developmental toxicity	Rodent	Studies in mice and rats report variable effects on fertility and sex hormone levels, with some evidence of reduced fertility and altered endocrine parameters. However, no maternal or developmental toxicity was observed in well-conducted prenatal studies.	Garg and Parasar 1965; Kiasalari et al. 2009; Belal et al. 2012; Prabu and Panchapakesan 2015
Asian ginseng	No effects expected No DART concern identified	No developmental or reproductive toxicity observed	Rodent, whole embryo culture	No developmental or reproductive effects were observed in multiple *in vivo* studies in rats and mice. Isolated constituents (e.g., ginsenoside Rb1) showed developmental effects in whole embryo culture, but these are not considered representative of typical exposure to the botanical extract.	Liu et al. 2005; Shin et al. 2010; NTP 2011; Kim et al. 2024
Blue cohosh	Developmental toxicity (teratogenic potential)	Cardiovascular and craniofacial malformations	Fish embryo, *in vitro* mechanistic data	Exposure to blue cohosh extracts disrupts cardiovascular and craniofacial development in fish embryos. Mechanistic studies indicate involvement of GATA2-EDN1 signaling and mitochondrial dysfunction, supporting its potential to induce developmental toxicity.	Satchithanandam et al. 2008; Wu et al. 2010; Datta et al. 2014
Comfrey	Developmental toxicity (teratogenic potential)	Malformations and perinatal toxicity	Rodent studies, mechanistic (genotoxicity)	Developmental toxicity, including malformations and perinatal effects, has been observed in rodent studies. Pyrrolizidine alkaloids are metabolically activated to reactive intermediates that induce DNA damage, providing mechanistic support for developmental hazard. EFSA has noted that it is not possible to determine whether these effects are secondary to maternal toxicity, introducing some uncertainty in interpretation.	Hirono et al. 1978; Mei et al. 2010; LiverTox 2012
Ephedra	Uncertain DART potential	Limited evidence of developmental effects; some malformations observed with isolated constituents	Avian embryo, livestock	Limited and inconsistent data are available. Developmental effects, including cardiac malformations, have been reported in chick embryo studies with ephedrine, while studies in livestock report maternal toxicity without clear developmental effects. Botanical preparations may lack measurable levels of active alkaloids in some cases, complicating interpretation. EFSA states lacks DART evidence.	Nishikawa et al. 1985; FDA 2008; LiverTox 2018
Green tea extract	Low DART concern	No developmental toxicity at typical exposure levels; effects at high doses	Rodent, zebrafish embryo, *in vitro*	Generally, no developmental or reproductive toxicity *in vivo*. High-dose exposures show effects on growth, viability, and cellular pathways including apoptosis and signaling disruption.	Isbrucker et al. 2006; Morita et al. 2009; Fan and Chan 2014; Hubbard et al. 2019; Wetmore et al. 2019
Goldenseal	Dose-dependent DART potential	Reduced fetal weight and developmental effects with constituent exposure	Rodent, zebrafish embryo, *in vitro* mechanistic	No developmental toxicity observed with whole extract *in vivo*; however, berberine shows dose-dependent developmental toxicity including fetal weight reduction and cardiac defects. Mechanistic data indicate ROS-mediated apoptosis.	Yao et al. 2005; Jahnke et al. 2006; Huang et al. 2020; Martini et al. 2020
Kava	Unknown DART potential	Limited data; no clear developmental endpoints	*In vitro* mechanistic	No direct DART studies available. Mechanistic data suggest neuronal activity effects including ion channel modulation, but relevance to DART remains unclear.	Merlin and Lindstrom 1992; Singh 1992; FDA 2020
Kratom	Developmental and reproductive toxicity potential	Malformations, mortality, behavioral and reproductive effects	Zebrafish embryo, rodent, *C. elegans*	Developmental toxicity including malformations and mortality observed in zebrafish embryos at high concentrations. Reproductive and behavioral effects reported in rodents and *C. elegans*.	Eggleston et al. 2019; Damodaran et al. 2021; Hughes et al. 2022; Zul Aznal et al. 2022; Jentsch and Pippin 2025
Milk thistle	Low or uncertain DART concern	No clear developmental toxicity; possible hormonal effects	Rodent	Limited data on DART endpoints. Some evidence of hormonal changes (e.g., prolactin), but no clear developmental or reproductive toxicity identified.	NTP n.d.; Capasso et al. 2009; EFSA 2011
*Usnea* Lichen	Developmental toxicity potential	Fetal toxicity and malformations	Rodent, zebrafish embryo	Usnic acid exposure results in fetal toxicity and morphological changes in rodents and zebrafish embryos, including antiangiogenic and teratogenic effects.	Draut et al. 2017; Silva et al. 2017
Yohimbe	Reproductive toxicity potential	Altered sperm parameters and male reproductive effects	Rodent	Studies in mice report changes in sperm count, motility, and reproductive organ weights, along with alterations in hormone levels and evidence of oxidative stress in testicular tissue. EFSA noted that no definitive conclusions can be drawn due to inconsistencies in the data, but findings may indicate potential effects on male fertility.	Al-Majed et al. 2006; EFSA 2013; NCCIH 2020
Tree tobacco	Developmental toxicity (teratogenic potential)	Craniofacial and limb malformations	Livestock, mechanistic	Teratogenic effects including limb defects and cleft palate observed in livestock. Linked to anabasine, which affects nicotinic acetylcholine receptors.	Keeler and Ward Crowe 1983; Welch et al. 2015
Rue	Developmental and reproductive toxicity	Embryo toxicity, fetal death, hormonal disruption	Zebrafish embryo, rodent, *in vitro*	Evidence of embryo toxicity, fetal death, and reproductive hormone disruption.	Gutiérrez-Pajares et al. 2003; de Freitas et al. 2005; Khouri and El-Akawi 2005; Forsatkar et al. 2018
Bitter melon	Developmental and reproductive toxicity	Malformations, fetal growth effects, and reproductive toxicity	Rodent, zebrafish embryo	Developmental effects, including malformations and fetal growth alterations, have been observed in rodents and zebrafish embryos. Reproductive toxicity, including effects on spermatogenesis and hormone levels, has also been reported. Findings vary by plant part and preparation, indicating dose- and extract-dependent effects.	Nwachi and McEwen 2009; Patil and Patil 2011; Adewale et al. 2014; Khan et al. 2019; Uche-Andriani et al. 2023
False hellebore	Developmental toxicity (teratogenic potential)	Craniofacial and skeletal malformations; embryonic death	Livestock, mechanistic	Developmental toxicity observed in livestock including craniofacial malformations and embryonic death. Effects are linked to steroidal alkaloids such as cyclopamine and jervine, which inhibit Hedgehog signaling.	Binns et al. 1965; James 1999; Panter et al. 2013
Poison hemlock	Developmental toxicity (teratogenic potential)	Skeletal malformations due to reduced fetal movement	Livestock, mechanistic	Teratogenic effects including limb and craniofacial defects observed in livestock. Mechanism involves activation of nicotinic acetylcholine receptors leading to reduced fetal movement.	Panter et al. 1985, 1988; Green et al. 2012; Green et al. 2013
Locoweed	Developmental and reproductive toxicity	Impaired fertility, embryo development, and neonatal health	Livestock, mechanistic	Extensive evidence in livestock demonstrates effects on both male and female reproductive function, including altered estrus cycles, reduced conception rates, impaired spermatogenesis, and effects on embryo and neonatal viability. Effects are attributed to swainsonine, which disrupts glycoprotein processing and cellular function, providing mechanistic support for reproductive and developmental toxicity.	Panter et al. 1999; Stegelmeier et al. 1999; Pfister et al. 2006
Cottonseed	Reproductive and developmental toxicity	Impaired fertility, embryonic development, and hormonal disruption	Rodent, livestock, human, mechanistic	Extensive evidence demonstrates reproductive toxicity in both males and females, including disrupted spermatogenesis, reduced sperm quality, altered estrous cycles, and impaired embryonic development. Effects are attributed to gossypol, which interferes with steroidogenesis and hormone regulation, with additional impacts on thyroid function.	Randel et al. 1992; Yu and Chan 1998; Waites et al. 1998; Wang et al. 2023

For each botanical, the presence or absence of evidence for DART effects is indicated, along with a description of DART endpoints, or other safety considerations relevant to each botanical. The type of evidence such as mechanistic studies, *in vivo* or livestock data is specified to provide context for the findings. Additional details highlight key study outcomes. Botanicals selected by other Working Groups are included here for completeness (shown in grey), and details of their review are provided in Supplemental File 1.

Botanical materials will be sourced from reputable suppliers with certificates of analysis, and characterized using established analytical methods (CEBS 2024; Waidyanatha et al. 2024). As detailed by Waidyanatha et al. (2024), characterization is essential to ensure consistency and reliability in safety assessments. Without it, variability in composition can hinder interpretation of toxicological results. In collaboration with the BSC Chemical Analysis Working Group, efforts will include determining extract solubility in assay-compatible solvents (e.g., ethanol, DMSO) and applying analytical techniques to confirm botanical identity and quantify key constituents.

## Ashwagandha

Ashwagandha (*Withania somnifera*), also known as winter cherry or Indian ginseng, is an evergreen shrub in the nightshade family and mainly grows in India, the Middle East, and Africa (Singh et al. 2011). It is commonly used as a rejuvenator and tonic (*Rasayana*) in Ayurvedic medicine. Generally, the root is used in herbal preparations.

An older study reported variable effects of root powder and extracts on fertility of rats and mice. Root powder (25 mg/day) administration to male and female mice for 10 days decreased the fertility rate by 25%, delayed mating by 1 day, and reduced litter size (Garg and Parasar 1965). Other studies found no toxic effects on reproduction or development in male and female rats. Pregnant rats fed ashwagandha during gestation days (GD) 5–19, critical periods of organogenesis and histogenesis, at 500, 1000, and 2000 mg/kg-day showed no maternal or fetal toxicity (Prabu and Panchapakesan 2015). Administration of ashwagandha root decoction (250 mg/kg) to male and female rats for eight months produced a healthier progeny compared to control (Sharma et al. 1985). Beneficial effects on male reproductive parameters have also been published (Kiasalari et al. 2009; Belal et al. 2012). Root powder (6.25%) in the diet for four weeks significantly raised serum progesterone, testosterone, and LH levels and significantly lowered serum FSH levels of male rats (Kiasalari et al. 2009). Similar effects on sex hormones in normal and diabetic rats fed root powder in the diet (6.25%) for four weeks were reported (Belal et al. 2012). Together, the data do not point to developmental concerns but are unclear on the hormonal and fertility effects of ashwagandha.

## Asian ginseng

Asian ginseng (*Panax ginseng*), a perennial herb with branching roots, is typically cultivated for 6–10 years before harvesting. This herb is indigenous to Asia, particularly in China and Korea. For thousands of years, the root of Asian ginseng has been used for medicinal purposes. According to the National Center for Complementary and Integrative Health (NCCIH), consuming ginseng orally for short periods (up to six months) is generally safe for most individuals (NCCIH 2020). The root extract of Asian ginseng was chosen for study by the BSC due to its reported safety across various endpoints.

The Division of Translational Toxicology [formerly Division of the National Toxicology Program (DNTP)] assessed Asian ginseng in rats and mice during short term (two-week) sub chronic (90 day) and two-year chronic studies but did not assess development (NTP 2011). In all treatment groups, duration, and species, the effects were either not different from controls or very minimal at the highest doses. Ovaries and testes were assessed and there were no differences from controls. Another study in rats dosed with Asian ginseng *via* feed found no effects in the F0, F1, or F2 generations, supporting that ginseng does not induce reproductive or developmental toxicity. Korean red ginseng enriched with ginsenosides was assessed in juvenile rats *via* oral gavage. There were no abnormalities in any treatment group compared to controls (Kim et al. 2024). A follow up uterotrophic bioassay to assess estrogenicity at postnatal days 19–21 and was negative (Kim et al. 2024). Mice exposed orally to 20, 200, or 2,000 mg/kg-day Korean red ginseng from two weeks before mating to gestational day 18 showed no developmental effects (Shin et al. 2010).

A study on the constituent ginsenoside Rb1 (DTXSID401316929) in whole mouse embryo culture reported effects on midbrain, forebrain, and optic systems (Liu et al. 2005). However, this compound has a very large molar mass (1109.307 g/mol) and is expected to have low permeability and low solubility (Liu et al. 2024). It is unlikely a developing embryo would be exposed to the same amount as an *in vitro* comparison. Based on multiple animal studies and long human usage, Asian ginseng is unlikely to exhibit developmental or reproductive effects.

## Bitter melon

Bitter melon (*Momordica charantia*, also known as bitter gourd) is a tropical and subtropical vine traditionally used in various medicinal systems, purported for its use in managing diabetes and digestive disorders. It is native to Asia, Africa, and the Caribbean, and continues to be used in both traditional and modern herbal medicine. Bitter melon contains several constituents, including charantin (a mixture of β-sitosteryl glucoside) and momordicin I (DTXSID501019962), believed to contribute to its pharmacological and biological effects. Notably, bitter melon is known in ethnomedicine to induce midterm abortion in humans, and Memorial Sloan Kettering advises avoiding its use during pregnancy due to animal studies indicating developmental abnormalities.

Several studies have looked at the potential for DART, with varying botanical parts and extract types. In zebrafish embryos, seed extract (but not fruit) exposure caused malformations in the absence of lethality, suggesting specific teratogenic activity rather than general toxicity (Khan et al. 2019). The fruit caused cardiac hypertrophy in the zebrafish embryo. A water extract of bitter melon fruit caused a higher incidence of resorption and malformations (particularly affecting reproductive organs) in Sprague Dawley rat fetuses and pups compared to controls (Uche-Nwachi and McEwen 2009). Pregnant Wistar rats exposed to a leaf extract of bitter melon during GD 6–15 (Trautenmuller et al. 2023) exhibited maternal toxicity (decreases in body mass gain and reduced water intake), but no effects on prenatal development. In contrast, a study using an ethanol extract of bitter melon fruit administered orally to pregnant mice from GD 6–17 showed a dose-dependent reduction in the average length of the fetal cranium and sternum (Andriani et al. 2023). Another study using a CO_2_ seed extract found no treatment-related effects when administered to pregnant Wistar rats from GD 5–19. However, this study is a preprint from 2022, not peer-reviewed, and it involved a different preparation method (Chung et al. 2022).

Reproductive effects have also been demonstrated in animal studies. In male albino mice, repeated intraperitoneal exposure to petroleum ether, chloroform, and ethanolic seed extracts led to reduced numbers of spermatogenic cells and increased androgen dependent organ weights, suggesting both antispermatogenic and androgenic activity, with the ethanolic extract reported to be most potent (Patil and Patil 2011). In adult female rats, oral administration of leaf extracts decreased circulating estrogen and progesterone levels, further supporting its impact on reproductive endocrinology (Adewale et al. 2014).

Taken together, the data across species and assay types consistently demonstrate that bitter melon has biological activity relevant to both male and female reproductive function. These findings, supported by both experimental studies and traditional use reports, indicate that bitter melon may pose developmental and reproductive risks and should be used with caution, particularly during pregnancy.

## Blue cohosh

Blue cohosh (*Caulophyllum thalictroides*) is an herbaceous perennial plant traditionally used in herbal medicine for various purposes, including stimulating the uterus and inducing labor or menstruation (Rader and Pawar 2013). While there have been reports of adverse effects associated with blue cohosh use during human pregnancy, such as fetal cardiac defects and acute nicotinic toxicity in the mother, these case reports are scarce and not well described in the literature.

Limited information is available on the potential mechanisms underlying the pharmacology of blue cohosh. Some researchers have proposed that certain alkaloids (e.g., quinolizidine type) and saponins (e.g., triterpene glycosides) present in blue cohosh may be responsible for the observed teratogenic and smooth muscle stimulant effects (Satchithanandam et al. 2008).

Studies using Japanese medaka (Oryzias latipes) and a methanolic extract of blue cohosh root, cardiovascular and craniofacial development was disrupted dose-dependently (Wu et al. 2010). Expression of genes involved in the GATA2-EDN1 signaling pathway, including gata2, ece1, and preproedn1, was increased in embryos exposed to blue cohosh. Mechanistic studies have also revealed that a methanolic extract disrupts cellular respiration and have provided insights attributing this effect to saponin constituents, which were shown to impair mitochondrial function by disrupting membrane integrity (Datta et al. 2014).

Based on the limited information published on its teratogenic effects, blue cohosh has the potential to exhibit developmental and reproductive effects.

## Cottonseed

The cotton plant (*Gossypium* spp.) is widely cultivated across tropical and subtropical regions worldwide. Traditionally, the plant has been prized primarily for its fiber, but cottonseed, a byproduct of cotton processing, has also found use as a feed ingredient and for cottonseed oil production. Cottonseed contains the naturally occurring polyphenolic aldehyde gossypol (DTXSID5023110), which exists in variable concentrations depending on the chemotype. Chinese chemotypes are particularly rich in gossypol and are associated with a higher risk of reproductive toxicity, while other varieties have been selectively bred to contain lower levels (Randel et al. 1992). Cottonseed has been extensively studied due to its known reproductive effects on animals and humans.

Gossypol has been identified as a potent reproductive toxicant in numerous species. In females, gossypol has been shown to disrupt estrous cycles, interfere with maintenance of pregnancy, and impair early embryo development. In males, gossypol causes infertility by disrupting spermatogenesis, reducing sperm motility, and decreasing sperm counts (Randel et al. 1992). The toxicological profile of gossypol has been characterized in human, rat, guinea pig, and bovine cell culture systems, where it has been found to inhibit several key steroidogenic enzymes (Yu and Chan 1998).

In China, gossypol was investigated as a potential male contraceptive. While initial studies suggested it was effective in reducing fertility, it became evident that the effects on spermatogenesis were often irreversible (Waites et al. 1998). Gossypol has also been identified as an androgen antagonist based on ToxCast COMPARA models, further supporting a mechanistic link to impaired male fertility.

Gossypol also impacts thyroid hormone metabolism. Studies in rats and sheep have shown that gossypol exposure can reduce serum concentrations of thyroxine (T4) and triiodothyronine (T3), though some variability in the direction of these effects has been reported (Gadelha et al. 2014). Histopathological examination of thyroid tissue from male rats revealed follicular degeneration and atrophy, along with pituitary thyrotropic cell hypertrophy, hyperplasia, and degranulation following gossypol administration (Gadelha et al. 2014).

Recent *in vivo* studies in mice have further demonstrated the reproductive and renal toxicity of gossypol. Male mice exposed to gossypol for 14 days exhibited testicular damage characterized by reduced seminiferous epithelial cells, atrophy of interstitial tissue, and incomplete differentiation of spermatogonia. These animals also showed impaired sperm acrosome development (Wang et al. 2023).

Overall, gossypol presents a consistent profile of reproductive toxicity across species and assay systems, raising concerns for both male and female fertility, with additional effects on thyroid function.

## False hellebore

False Hellebore (*Veratrum californicum*) is a poisonous flowering plant of the buttercup family that grows in the western United States and is a known developmental toxicant primarily affecting livestock such as sheep, cattle, and goats (Panter et al. 2013).

Livestock which have been exposed to false hellebore, either through grazing or experimentally, give birth to offspring with various birth defects such as craniofacial malformations (i.e., cyclopia, cleft palate), limb defects, tracheal stenosis, or undergo embryonic death (Binns et al. 1965; James 1999; Welch et al. 2009; Panter et al. 2013). The discovery of the effects of false hellebore on grazing livestock has led to investigations into the constituents responsible for these defects. Notable components include cyclopamine (DTXSID6043709) and jervine (DTXSID70895026), which are part of the jervanine class of alkaloid steroids and are teratogens (Panter et al. 2013). Of these, cyclopamine has sparked interest in research and therapeutic development due to its ability to inhibit the Hedgehog signaling (HH) pathway, a key developmental pathway that can become aberrantly expressed in some cancers (James et al. 2004; Panter et al. 2013; Lee et al. 2014; Li et al. 2016). Jervine is also a Hedgehog signaling pathway inhibitor that has gained some interest in research as a potential antifungal (Kubo et al. 2022), anti-inflammatory (Dumlu et al. 2019), and anti-tumor agent (Lei and Huo 2020). Inhibition of Hedgehog pathway signaling is *via* binding to and antagonizing the Smoothened receptor, a pivotal transmembrane protein in the Hedgehog signaling pathway (Chen et al. 2002).

Finally, if false hellebore is ingested by people, it can cause bradycardia and hypotension, nausea, and vomiting. These effects are attributed to the cardiotoxic actions of veratramine and cevanine-type alkaloids, which alter sodium channel function (Jaffe et al. 1990).

Overall, while false hellebore is expected to induce developmental effects, available evidence of teratogenicity is largely limited to ruminants following oral exposure. This specificity is believed to result from acid hydrolysis of an ether bond at low pH, and thus, teratogenicity is less likely to occur in monogastric species such as humans. Although the likelihood of human developmental toxicity may be lower, further testing is warranted to fully assess potential risks.

## Goldenseal

Goldenseal (*Hydrastis canadensis* L.) is a medicinal plant traditionally used by Native Americans as a coloring agent and as a medicinal remedy for common diseases and conditions like wounds, digestive disorders, ulcers, skin and eye ailments, and cancer. Goldenseal is a rich source of alkaloid phytochemicals. Berberine (DTXSID9043857) is one of the most biologically active alkaloids identified in goldenseal and has been purported for its anti-microbial, anti-inflammatory, hypolipidemic, hypoglycemic, and antioxidant properties. There are few studies of the developmental and reproductive toxicity of goldenseal and related extracts. This botanical was selected for its known botanical-drug interactions by the Hepatotoxicity Working Group in the BSC.

Studies evaluated the developmental and reproductive toxicity of berberine chloride dihydrate in rats and mice *via* diet or gavage (Jahnke et al. 2006). In rats and mice, developmental toxicity LOELs were 1,000 mg/kg-day and 841 mg/kg-day, respectively, both based on reduced fetal body weight. However, a study on goldenseal developmental toxicity in pregnant rats found no increase in pre- or post-implantation losses, fetal body weight, or incidence of malformations when administered at 65 times the human dose during gestation (Yao et al. 2005). Yet, goldenseal induced toxicity in rat embryos cultured *in vitro*, suggesting poor absorption *in vivo* might account for the lack of observed toxicity in live animals.

Interestingly, berberine at low doses (2.5 μM *in vitro* and 1 mg/kg *in vivo*) enhanced oocyte maturation, *in vitro* fertilization (IVF) rates, and subsequent embryonic development by decreasing intracellular reactive oxygen species (ROS) levels and preventing apoptosis (Huang et al. 2020). Conversely, higher doses (10 μM *in vitro* and 5 mg/kg *in vivo*) impaired oocyte maturation, reduced IVF rates, and induced apoptosis in early-stage embryos by increasing ROS production. These findings suggest that berberine’s effects on embryonic development are mediated through ROS-triggered, caspase-3-dependent apoptotic pathways, highlighting its dual role as either a protective antioxidant or a harmful pro-oxidant depending on the dose (Huang et al. 2020). Berberine has also been shown to induce developmental toxicity in zebrafish embryos in a time- and concentration-dependent manner (Martini et al. 2020). Treated embryos exhibit cardiac looping defects, abnormal heart morphology, atrial endocardial/myocardial detachment, and impaired angiogenesis.

Together, the results suggest that goldenseal has the potential for developmental toxicity mediated through ROS-triggered, caspase-3-dependent apoptotic pathways. However, further pharmacokinetic studies are needed to confirm this conclusion.

## Locoweeds

Locoweeds are plants from the *Astragalus* and *Oxytropis* genera. They are native to North America and Asia. Locoweeds are well-known for their toxicity to livestock in arid and mountainous grazing regions. These plants contain swainsonine (DTXSID5046356), an indolizidine alkaloid responsible for their toxicity (Molyneux and James 1982). Traditionally, locoweeds have not been used for medicinal purposes but are of significant concern in veterinary toxicology due to their impact on animal health.

Evidence related to DART is extensive and primarily comes from animal studies. In female livestock, locoweed ingestion affects numerous aspects of reproduction, including estrus behavior, cycle length, ovarian function, conception rates, and fetal viability (Panter et al. 1999). Experimental feeding studies in sheep and cattle have demonstrated altered ovarian function, delayed estrus, and reduced conception rates after consuming *Astragalus mollissimus*, *A. lentiginosus*, and *Oxytropis sericea*. Cycling ewes fed locoweed at 10–15% of their diet for 20 days showed shortened estrus behavior and fewer viable embryos. In mature cows, exposure resulted in prolonged estrous cycles and, at high doses, ovarian cysts, which resolved once feeding ceased (Panter et al. 1999).

Swainsonine has also been shown to impact post-implantation development. Although *in vitro* studies using bovine embryos suggest that swainsonine does not directly affect oocyte maturation or early embryo development (Wang et al. 1999), *in vivo* studies indicate that maternal toxicity may impair uterine or placental function. Histological evidence from swainsonine-treated animals supports this, with lesions observed in high-accumulating tissues like liver and kidney (Stegelmeier et al. 1999). Locoweed ingestion during mid-gestation disrupted maternal–infant bonding in sheep and resulted in weaker, less vigorous lambs with impaired suckling behavior (Pfister and Price 1996). Swainsonine is also excreted in milk, potentially leading to continued exposure in neonates (James and Hartley 1977).

Male reproductive function is also adversely affected by locoweed exposure. In rams, ingestion of *A. lentiginosus* led to histological degeneration of the seminiferous epithelium, reduced sperm motility, and increased abnormal sperm morphology, including retained cytoplasmic droplets and kinked tails (Panter et al. 1989). Although some effects reversed after exposure ceased, studies reported long-term impacts on behavior and fertility. For example, in a 60-day trial, rams showed reduced libido and neurological signs such as tremors and proprioceptive deficits, with subsequent reductions in conception rates even before semen quality changed (Wang et al. 1998). Persistent neurological damage was observed in several animals. Another study found reduced scrotal circumference and testosterone response to GnRH 35 days after feeding *O. sericea* had stopped, indicating delayed effects on testicular function (Ortiz et al. 1997).

Taken together, the breadth and consistency of animal data clearly indicate that locoweeds disrupt both male and female reproductive systems, with effects on fertility, embryo development, and neonatal health. The mechanism of action *via* swainsonine inhibition of α-mannosidase and glycoprotein processing adds biological plausibility to these observations (Elbein et al. 1981; Li et al. 2012).

## Milk thistle

Milk thistle (*Silybum marianum*) is an annual plant in the daisy family, originally from Southern Europe, Asia Minor, and Northern Africa, and has spread to North and South America and Australia. Herbalists and naturopathic doctors use milk thistle fruit to treat digestive issues, while some medical professionals use plant-based formulations to address liver damage. The plant’s fruits contain a variety of flavonolignans, such as silybin (a mixture of two diastereomers, silybin A and silybin B, in approximately equimolar ratio) (Kroll et al. 2007). These constituents and others combine to form silymarin, which represents about 70% of the content in milk thistle extract (Graf et al. 2007; Kroll et al. 2007; Shibano et al. 2007).

Milk thistle is generally considered safe and was selected as a pan-negative across the BSC working groups. One strong line of evidence is its reported non-toxicity, as evidenced by a two-year study conducted by the NTP on rats and mice, however, they did not assess developmental endpoints (NTP n.d.). Reproductive organs were not affected. Most studies focus on the proposed protective properties of milk thistle or their constituents. One study pointed to mycotoxin content leading to potential toxic constituents in milk thistle (Boško et al. 2022).

A silymarin extract (25 to 200 mg/kg per day) given orally for 14 days to lactating Wistar female rats was reported to increase serum prolactin levels (Capasso et al. 2009). However, an EFSA panel rejected a claim that a milk thistle extract leads to increased production of breast milk after delivery in humans due to shortcomings in the study (EFSA 2011). NCCIH says ‘Little is known about whether it’s safe to use milk thistle during pregnancy or while breastfeeding’.

Generally, milk thistle and its constituents are not expected to be developmental and/or reproductive toxicants, but there are not sufficient studies to rule out effects.

## Poison hemlock

Poison hemlock (*Conium maculatum*), infamous in the death of Socrates and the works of Shakespeare, is among the most toxic plants. Ingestion of poison hemlock has resulted in acute toxicity and death in several animal species (e.g., cattle, horses, pigs, sheep, chickens, and turkeys) as well as humans (Vetter 2004). A member of the carrot family (Apiaceae, formerly Umbelliferae), poison hemlock is native to Europe and western Asia, but it is found as a common weed in Europe, North America, South America, North Africa, Australia, and New Zealand (Vetter 2004). The piperidine alkaloids, coniine (DTXSID301019378) and γ-coniceine (DTXSID00166872), are primarily responsible for the toxicity of poison hemlock and are distributed throughout the plant (López et al. 1999).

In addition to its acute toxicity characterized by neuromuscular inhibition leading to respiratory paralysis, poison hemlock can be teratogenic when ingested during pregnancy. Teratogenic effects of poison hemlock consist of skeletal malformations with observations of cleft palate, twisted or bowed limbs (arthrogryposis), twisted or bowed spine (scoliosis), and twisted neck (torticollis) in pigs (Panter et al. 1985), goats (Panter et al. 1990), and cattle (Keeler and Balls 1978). A reduction in fetal movement was observed in sheep following the feeding of dams with 5–10 g fresh ground poison hemlock per kg body weight (Panter et al. 1988). Panter et al. (1988) proposed that reduced fetal movement was the underlying cause of observed skeletal malformations, with the duration of restriction corresponding to the severity and permanence of associated malformations (Panter et al. 1988).

The mechanism of action for both the acute and teratogenic effects of poison hemlock involves activation of nicotinic acetylcholine receptors (nAChR) by the piperidine alkaloids (Green et al. 2013). The piperidine alkaloids induce persistent activation of fetal muscle type nAChRs, which leads to desensitization of the receptors, resulting in decreased fetal movement and accompanying malformations (Green et al. 2012). Overall, poison hemlock is expected to be developmentally toxic.

## Rue

Rue (*Ruta graveolens*) is a perennial herb native to the Mediterranean with a long history of medicinal use and, more so in former times, culinary use. Traditionally, it was used to stimulate menstruation, induce abortion, and treat ailments like cramps and rheumatism, but it has notable developmental and reproductive toxic effects, supported by historical and experimental evidence. Furanocoumarins isolated from rue, such as chalepensin (DTXSID90157153) have been implicated in these effects.

In superovulated mice, oral administration of aqueous rue extracts at concentrations of 10 and 20% for four days resulted in a high proportion of abnormal embryos (37 and 63%, respectively) (Gutiérrez-Pajares et al. 2003). The highest dose also caused a reduction in cell numbers per embryo and a slight delay in preimplantation embryo transport. However, in a separate study, administration of rue extract during preimplantation, implantation, and post-implantation phases did not significantly affect these stages nor exhibit estrogenic activity (as measured by uterine weight or premature vaginal opening), although it was associated with fetal death (de Freitas et al. 2005). A review article cited a rat study where rue extracts increased the number of resorbed embryos and a hexane extract specifically caused significant fetal weight reduction, alongside maternal and fetal toxicity (Luo et al. 2024).

Research using *C. elegans* demonstrated that exposure to rue impaired reproduction rates; nematodes initially showed slowed growth but later adapted to survive the toxic environment (Moges et al. 2023). In zebrafish, treatment with rue led to reduced egg production and fertilization rates, accompanied by decreased estradiol-17β (E2) levels in females, lower testosterone (T) levels in both sexes, and a decline in thyroid hormones (T3 and T4) (Forsatkar et al. 2018).

Traditionally, in Persian medicine, rue has been used as a male contraceptive, and various studies have explored its effects on male fertility. For example, a study in mice reported that a 300 mg/kg dose reduced sperm number and viability while increasing the percentage of immature sperm and DNA fragmentation (Ebadi Manas and Najafi 2019). Oral administration of aqueous rue extract (500 mg/kg) to male rats over 60 days decreased reproductive organ weight, sperm motility and density, and serum testosterone and follicle-stimulating hormone (FSH) levels. The extract also suppressed sexual and aggressive behaviors, resulting in fewer impregnated females, reduced implantation sites, and lower numbers of viable fetuses (Khouri and El-Akawi 2005).

*In vitro*, a 1:1 aqueous extract of rue mixed with fresh human sperm caused immediate sperm immobilization, likely due to potassium channel blockade. Importantly, sperm viability, DNA integrity, and mitochondrial activity were unaffected, and ∼30% of sperm regained motility after washing (Harat et al. 2008). Additionally, ethanol extracts of rue and *Cannabis sativa* administered intraperitoneally to male rats at 20 mg/day for 20 days significantly reduced epididymal sperm counts, with rue exhibiting a stronger inhibitory effect on spermatogenesis than Cannabis (Sailani and Moeini 2007).

Overall, rue is expected to induce development and reproductive effects.

## Tree tobacco

Tree tobacco (*Nicotiana glauca)* is an evergreen shrub native to South America, but also is widespread in other continents, including as a common weed in the southwest United States. Tree tobacco is known to induce cleft palate and skeletal malformations in multiple species, including pigs, cows, goats, and sheep (Panter et al. 1990). Pregnant pigs fed *Nicotiana glauca* had piglets with birth defects, including limb deformities and cleft palates. Limb issues occurred at any point during gestation, while cleft palates appeared only when the plant was eaten before day 35 (Keeler and Ward Crowe 1983). These teratogenic effects have been linked to the inhibition of fetal movement caused by anabasine exposure during development. Anabasine (DTXSID9041607) and related substances are piperidine alkaloids that act as an agonist toward the nicotinic acetylcholine receptor (nAChR) (Welch et al. 2015; Xing et al. 2020).

Given the strong evidence in animals and mechanistic information, tree tobacco is expected to induce developmental toxicity.

## *Usnea* lichen

*Usnea* is a genus of lichen that grows on trees, bushes, rocks, and soil of temperate and humid climates worldwide (Prateeksha et al. 2016). Lichens are organisms that arise from a mutualistic relationship between algae or cyanobacteria with fungi. *Usnea* has long been used in traditional medicine to treat urinary ailments, wounds, and inflammation (Sepahvand et al. 2021). Today, it is commonly used to aid in weight loss, promote wound healing, and protect against certain cancers. Studies show that usnic acid (DTXSID0040123) is the active compound present in *Usnea* species extracts, a compound which has a high antibacterial and cytotoxic activity (Sepahvand et al. 2021).

Extracts of *Usnea* have been considered safe, yet high doses have been associated with reversible liver injury (Guo et al. 2008). Further, studies have supported anti-mutagenic effects related to the antioxidant activity of *Usnea* sp. To evaluate the effects of usnic acid on organogenesis female rats were treated with 15 or 20 mg/kg *via* oral gavage from GD 6–15. On GD 20, lower weight gain during pregnancy, increased resorptions, fewer viable fetuses, and lower fetal weight were observed. The fetuses displayed morphological changes in the eyes and limbs, a reduction in megakaryocytes, and an increased number of hepatocytes (Silva et al. 2017). Further, usnic acid displayed antiangiogenic and teratogenic effects (curvature of spine, reduced body length, and yolk sac sizes in zebrafish embryos at 24 hpf (Draut et al. 2017). *In vitro*, usnic acid was markedly cytotoxic and interfered with MMP2 expression in 518A2 melanoma cells. These studies suggest a potential for *Usnea* to have developmental effects.

## Common foods as negative controls for DART

Given the sparsity of botanicals with clear evidence of no DART concern, common fruits and vegetables such as (but not limited to) cabbage (Brassica oleracea var. capitata leaf), sweet orange (Citrus sinensis fruit), carrot (Daucus carota root), strawberries (Fragaria x ananassa fruit), and cauliflower (Brassica oleracea var. botrytis flower head) may also be considered as negative controls. These widely consumed foods have well-established safety profiles and lack evidence of adverse effects on reproduction or development, making them ideal for comparison to botanicals with uncertain or suspected toxicities. Bioactivity profiling using the BioMAP system revealed that common fruits and vegetables exhibit broad yet low-potency bioactivity signatures, reflecting their complex phytochemical composition without eliciting significant toxicological responses (Wetmore et al. 2019). This contrasts with other botanical or isolated chemical constituents, which often demonstrate higher potency or more specific bioactivity in high-throughput screening platforms such as those employed by the Tox21 program (Hubbard et al. 2019). Consequently, incorporating these common items as additional negative controls provides the means to contextualize and interpret NAM data from botanicals under investigation.

## Conclusions and way forward

The DART potential of many botanicals is not well understood. The BSC aims to explore the use of NAMs for screening botanical extracts, and this strategy paper outlines the assays and botanicals chosen by the DART Working Group, a diverse team of experts, for that purpose. Assays include biomarker, functional, as well as mechanistic NAMs that will be explored collectively to contribute to a more comprehensive DART assessment strategy, rather than focusing on a single test system alone. This will allow us to target key mechanisms of DART mode of action, expand biological coverage beyond known mechanisms, and integrate across biological levels. Eighteen botanicals in total have been reviewed, spanning no DART effects, unknown or suspected effects, as well as clear evidence of developmental or reproductive effects. The next steps involve testing a number of these botanicals and determining how the data generated can be considered in screening for DART, compared to those reported in the literature.

At present, there is no universally accepted decision framework for integrating data from multiple NAMs to guide subsequent actions in developmental and reproductive toxicity (DART) assessment, particularly for complex mixtures such as botanicals. Selection and interpretation of assays are expected to be driven by problem formulation and context of use, including the specific DART endpoints of interest, life stage considerations, exposure scenarios, and available supporting data. Consideration of exposure and dose relevance is also critical for contextualizing NAM findings and can be supported through integration of use patterns, estimated human intake, and comparison of bioactive concentrations to expected internal exposure levels (e.g., *via* IVIVE where feasible). Where sufficient data are available, physiologically based pharmacokinetic (PBPK) modeling can further support translation of *in vitro* bioactivity into estimates of tissue or plasma concentrations, providing additional context for interpreting NAM findings in relation to human exposure. As such, the approach described here is not intended to be prescriptive, but rather to illustrate a flexible battery of complementary models. The development of standardized, fit-for-purpose decision criteria for integrating NAM data in DART assessment remains an important area for future research.

This manuscript is envisioned by the DART Working Group to be the first in a series of papers reporting the outcome. Future papers will report on the data and how it can be used for several case studies to determine the DART risk of botanical extracts.

While the application of NAMs continues to advance, challenges remain in their broader implementation, including regulatory acceptance and integration of diverse data streams into coherent decision-making frameworks. At the same time, these challenges represent a significant opportunity, particularly for products such as dietary supplements, which often lack extensive premarket toxicity testing, and cosmetics, where animal testing is restricted or prohibited in some jurisdictions. In these contexts, NAMs offer a practical and scalable approach to generate relevant safety data and support more informed risk assessment.

## Supplementary Material

Supplemental 2.docx

Supplemental.docx

## Data Availability

Tables 2 and 3 are also available for download in the Supplemental Files. Chemical Analysis information for the botanicals can be found at https://doi.org/10.22427/NTP-DATA-500-007-001-000-3.
