# Supplementary material for: A screening strategy for identifying the developmental and reproductive toxicity potential of botanicals
Source: Pharm Biol. 2026 Apr 28;64(1):639–67. doi: 10.1080/13880209.2026.2659421 (PMC13126950; doi:10.1080/13880209.2026.2659421)
Supplement: Supplemental 2.docx [file IPHB_A_2659421_SM3746.docx]

Table 3: List of botanicals, including their standardized common and scientific names, and the part(s) of the plant used to derive the botanical extract. Botanicals selected by the DART Working Group based on their DART profiles are in bold.

| Standardized Common Name | Scientific Name | Plant Part(s) |
| --- | --- | --- |
| Aristolochia fangchi | *Aristolochia fangchi Y.C. Wu ex L.D. Chou & S.M. Hwang* | Root |
| **Ashwagandha** | ***Withania somnifera (L.) Dunal*** | **Root** |
| **Asian Ginseng** | ***Panax ginseng C.A. Mey.*** | **Root** |
| **Bitter Melon** | ***Momordica charantia* L.** | **Seed & Fruit** |
| **Blue Cohosh** | ***Caulophyllum thalictroides* (L.) Michx.** | **Root & Rhizome** |
| Comfrey | *Symphytum officinale* L. | Root |
| **Cottonseed** | ***Gossypium* spp.** | **Seed** |
| Ephedra | *Ephedra sinica Stapf* | Aerial Parts |
| **False Hellebore** | ***Veratrum album*** L.**, *Veratrum californicum*** L. | Root & Rhizome |
| **Goldenseal** | ***Hydrastis canadensis* L.** | **Root & Rhizome** |
| Green Tea | *Camellia sinensis* (L.) Kuntze | Leaf |
| Kava | *Piper methysticum* G.Forst. | Root & Rhizome |
| Kratom | *Mitragyna speciosa* (Korth.) Havil. | Leaf |
| **Locoweed** | ***Astragalus* spp*.; Oxytropis* spp.** | **Aerial Parts** |
| **Milk Thistle** | ***Silybum marianum* (L.) Gaertn.** | **Seed** |
| **Poison Hemlock** | ***Conium maculatum* L.** | **Whole Plant** |
| **Rue** | ***Ruta graveolens* L.** | **Leaf** |
| **Tree Tobacco** | ***Nicotiana glauca* Graham** | **Leaf** |
| **Usnea** | ***Usnea* spp.** | **Whole Lichen** |

Table 4. Summary of selected botanicals for developmental and reproductive toxicity (DART) evaluation. For each botanical, the presence or absence of evidence for DART effects is indicated, along with a description of DART endpoints, or other safety considerations relevant to each botanical. The type of evidence such as mechanistic studies, in vivo or livestock data is specified to provide context for the findings. Additional details highlight key study outcomes. Botanicals selected by other Working Groups are included here for completeness (shown in grey), and details of their review are provided in Supplemental File 1.

| Botanical (Common Name) | Evidence for DART? | Evidence or lack thereof of DART phenotype | Evidence type | Details | Key References |
| --- | --- | --- | --- | --- | --- |
| Aristolochia fangchi | Developmental toxicity (teratogenic potential) | Malformations and developmental neurotoxicity | Zebrafish embryo, mechanistic (genotoxicity) | Zebrafish studies show developmental nephrotoxicity, neurotoxicity, and malformations. Aristolochic acids are well-characterized genotoxicants associated with human carcinogenicity, providing mechanistic support for developmental hazard. | Debelle et al., 2008; Nortier & Vanherweghem, 2002; Phillips & Arlt, 2009; Ding & Chen, 2012; Chen et al., 2021 |
| Ashwagandha | Uncertain DART potential | Mixed effects on fertility and hormonal endpoints; no evidence of developmental toxicity | Rodent | Studies in mice and rats report variable effects on fertility and sex hormone levels, with some evidence of reduced fertility and altered endocrine parameters. However, no maternal or developmental toxicity was observed in well-conducted prenatal studies. | Garg & Parasar, 1965; Prabu & Panchapakesan, 2015; Belal et al., 2012; Kiasalari et al., 2009 |
| Asian ginseng | No effects expected  No DART concern identified | No developmental or reproductive toxicity observed | Rodent,  whole embryo culture | No developmental or reproductive effects were observed in multiple in vivo studies in rats and mice. Isolated constituents (e.g., ginsenoside Rb1) showed developmental effects in whole embryo culture, but these are not considered representative of typical exposure to the botanical extract. | NTP, 2011; Kim et al., 2024; Shin et al., 2010; Liu et al., 2005 |
| Blue cohosh | Developmental toxicity (teratogenic potential) | Cardiovascular and craniofacial malformations | Fish embryo, in vitro mechanistic data | Exposure to blue cohosh extracts disrupts cardiovascular and craniofacial development in fish embryos. Mechanistic studies indicate involvement of GATA2-EDN1 signaling and mitochondrial dysfunction, supporting its potential to induce developmental toxicity. | Wu et al., 2010; Datta et al., 2014; Satchithanandam et al., 2008 |
| Comfrey | Developmental toxicity (teratogenic potential) | Malformations and perinatal toxicity | Rodent studies, mechanistic (genotoxicity) | Developmental toxicity, including malformations and perinatal effects, has been observed in rodent studies. Pyrrolizidine alkaloids are metabolically activated to reactive intermediates that induce DNA damage, providing mechanistic support for developmental hazard. EFSA has noted that it is not possible to determine whether these effects are secondary to maternal toxicity, introducing some uncertainty in interpretation. | LiverTox, 2012; Mei et al., 2010; FDA, 2001; Hirono et al., 1978; EFSA, 2011a |
| Ephedra | Uncertain DART potential | Limited evidence of developmental effects; some malformations observed with isolated constituents | Avian embryo, livestock | Limited and inconsistent data are available. Developmental effects, including cardiac malformations, have been reported in chick embryo studies with ephedrine, while studies in livestock report maternal toxicity without clear developmental effects. Botanical preparations may lack measurable levels of active alkaloids in some cases, complicating interpretation. EFSA states lacks DART evidence. | FDA, 2008; LiverTox, 2018; Nishikawa et al., 1985; Keeler, 1989; EFSA Panel (Additives & Food), 2013 |
| Green tea extract | Low DART concern | No developmental toxicity at typical exposure levels; effects at high doses | Rodent, zebrafish embryo, in vitro | Generally, no developmental or reproductive toxicity in vivo. High-dose exposures show effects on growth, viability, and cellular pathways including apoptosis and signaling disruption. | Morita et al., 2009; Isbrucker et al., 2006; Fan & Chan, 2014; Zhang et al., 2023; Barenys et al., 2017; Wetmore et al., 2019; Hubbard et al., 2019 |
| Goldenseal | Dose-dependent DART potential | Reduced fetal weight and developmental effects with constituent exposure | Rodent, zebrafish embryo, in vitro mechanistic | No developmental toxicity observed with whole extract in vivo; however, berberine shows dose-dependent developmental toxicity including fetal weight reduction and cardiac defects. Mechanistic data indicate ROS-mediated apoptosis. | Jahnke et al., 2006; Yao et al., 2005; Huang et al., 2020; Martini et al., 2020 |
| Kava | Unknown DART potential | Limited data; no clear developmental endpoints | In vitro mechanistic | No direct DART studies available. Mechanistic data suggest neuronal activity effects including ion channel modulation, but relevance to DART remains unclear. | Merlin & Lindstrom, 1992; Singh, 1992; Pittler & Ernst, 2000; FDA, 2020; Humberston et al., 2003; Stickel & Shouval, 2015; Soares et al., 2022 |
| Kratom | Developmental and reproductive toxicity potential | Malformations, mortality, behavioral and reproductive effects | Zebrafish embryo, rodent, *C. elegans* | Developmental toxicity including malformations and mortality observed in zebrafish embryos at high concentrations. Reproductive and behavioral effects reported in rodents and C. elegans. | EUDA, 2024; Eggleston et al., 2019; Jentsch & Pippin, 2025; Zul Aznal et al., 2022; Damodaran et al., 2021; Hughes et al., 2022 |
| Milk thistle | Low or uncertain DART concern | No clear developmental toxicity; possible hormonal effects | Rodent | Limited data on DART endpoints. Some evidence of hormonal changes (e.g., prolactin), but no clear developmental or reproductive toxicity identified. | NTP; Capasso et al., 2009; EFSA, 2011 |
| Usnea Lichen | Developmental toxicity potential | Fetal toxicity and malformations | Rodent, Zebrafish embryo | Usnic acid exposure results in fetal toxicity and morphological changes in rodents and zebrafish embryos, including antiangiogenic and teratogenic effects. | Silva et al., 2017; Draut et al., 2017 |
| Yohimbe | Reproductive toxicity potential | Altered sperm parameters and male reproductive effects | Rodent | Studies in mice report changes in sperm count, motility, and reproductive organ weights, along with alterations in hormone levels and evidence of oxidative stress in testicular tissue. EFSA noted that no definitive conclusions can be drawn due to inconsistencies in the data, but findings may indicate potential effects on male fertility. | NCCIH, 2020; Al-Majed et al., 2006; EFSA (Chain), 2013 |
| Tree tobacco | Developmental toxicity (teratogenic potential) | Craniofacial and limb malformations | Livestock, mechanistic | Teratogenic effects including limb defects and cleft palate observed in livestock. Linked to anabasine, which affects nicotinic acetylcholine receptors. | Keeler & Ward Crowe, 1983; Welch et al., 2015 |
| Rue | Developmental and reproductive toxicity | Embryo toxicity, fetal death, hormonal disruption | Zebrafish, rodent, in vitro | Evidence of embryo toxicity, fetal death, and reproductive hormone disruption. | Gutiérrez-Pajares et al., 2003; de Freitas et al., 2005; Khouri & El-Akawi, 2005; Forsatkar et al., 2018 |
| Bitter melon | Developmental and reproductive toxicity | Malformations, fetal growth effects, and reproductive toxicity | Rodent, zebrafish embryo | Developmental effects, including malformations and fetal growth alterations, have been observed in rodents and zebrafish embryos. Reproductive toxicity, including effects on spermatogenesis and hormone levels, has also been reported. Findings vary by plant part and preparation, indicating dose- and extract-dependent effects. | Khan, 2019; Uche-Nwachi, 2009; Andriani et al., 2023; Patil & Patil, 2011; Adewale, 2014 |
| False hellebore | Developmental toxicity (teratogenic potential) | Craniofacial and skeletal malformations; embryonic death | Livestock, mechanistic | Developmental toxicity observed in livestock including craniofacial malformations and embryonic death. Effects are linked to steroidal alkaloids such as cyclopamine and jervine, which inhibit Hedgehog signaling. | Panter et al., 2013; Binns et al., 1965; James, 1999 |
| Poison hemlock | Developmental toxicity (teratogenic potential) | Skeletal malformations due to reduced fetal movement | Livestock, mechanistic | Teratogenic effects including limb and craniofacial defects observed in livestock. Mechanism involves activation of nicotinic acetylcholine receptors leading to reduced fetal movement. | Panter et al., 1985; Panter et al., 1988; Green et al., 2012; Green et al., 2013 |
| Locoweed | Developmental and reproductive toxicity | Impaired fertility, embryo development, and neonatal health | Livestock, mechanistic | Extensive evidence in livestock demonstrates effects on both male and female reproductive function, including altered estrus cycles, reduced conception rates, impaired spermatogenesis, and effects on embryo and neonatal viability. Effects are attributed to swainsonine, which disrupts glycoprotein processing and cellular function, providing mechanistic support for reproductive and developmental toxicity. | Panter et al., 1999b; Panter et al., 1999c; Pfister et al., 2006; Stegelmeier et al., 1998 |
| Cottonseed | Reproductive and developmental toxicity | Impaired fertility, embryonic development, and hormonal disruption | Rodent, livestock, human, mechanistic | Extensive evidence demonstrates reproductive toxicity in both males and females, including disrupted spermatogenesis, reduced sperm quality, altered estrous cycles, and impaired embryonic development. Effects are attributed to gossypol, which interferes with steroidogenesis and hormone regulation, with additional impacts on thyroid function. | Randel et al., 1992; Yu & Chan, 1998; Waites et al., 1998; Wang et al., 2023 |
