# Supplementary material for: A screening strategy for identifying the developmental and reproductive toxicity potential of botanicals
Source: Pharm Biol. 2026 Apr 28;64(1):639–67. doi: 10.1080/13880209.2026.2659421 (PMC13126950; doi:10.1080/13880209.2026.2659421)
Supplement: Supplemental.docx [file IPHB_A_2659421_SM3745.docx]

## **Aristolochia fangchi**

## *Aristolochia fangchi*, commonly known as *fang ji* or *guang fang ji* among other names, is a perennial climbing vine. This plant was at the center of a notorious kidney disease outbreak in Belgium during the 1990s (Debelle et al., 2008; Nortier & Vanherweghem, 2002). The incident involved patients using a weight-loss supplement intended to contain *Stephania tetrandra* S.Moore, *Menispermaceae* (known as stephania or *fen fang ji*), but mistakenly formulated with Aristolochia fangchi root extracts due to mix-ups with similar common names. Within three years, over 100 individuals who inadvertently consumed Aristolochia fangchi suffered from kidney damage, with many experiencing kidney failure that necessitated transplants or dialysis. Additionally, some of these patients developed cancer or urinary tract disorders. Aristolochia fangchi and related species produce aristolochic acids, including aristolochic acid (DTXSID0040969) and II (DTXSID00197166), which are recognized for their genotoxic and nephrotoxic properties. This particular botanical was chosen for investigation due to its genotoxic effects.

Since aristolochia is known to be genotoxicity, there could be developmental effects. Genotoxic agents can directly impact developmental processes by damaging the DNA of rapidly dividing cells in a developing embryo or fetus (Phillips & Arlt, 2009). Such DNA damage can lead to mutations or disruptions in gene expression that are critical for normal development, potentially causing congenital abnormalities, developmental delays, or malformations. The timing of exposure is crucial, as damage during critical periods of organ formation can have severe consequences, including irreversible defects. One study evaluated the developmental nephrotoxicity of aristolochic acid in zebrafish embryos, which had a concentration-dependent increase in both kidney and other developmental malformations (Ding & Chen, 2012). Another study that looked at non-teratogenic concentrations in zebrafish found larval hyperactivity, effects on ocular development, and neurotoxicity (Chen et al., 2021).

Overall, it is unknown whether *Aristolochia fangchi* will induce DART, but there is some evidence that it can via genotoxic mechanisms.

**Comfrey**

Comfrey (*Symphytum*), originally from Asia and Europe, is a genus of flowering plants with oval to lance-shaped leaves and bell-shaped flowers. Its root has been used in medicine for more than 2000 years, for various ailments including broken bones, lung congestion, and joint inflammation, and to promote wound healing (LiverTox, 2012). However, it contains genotoxic compounds known as pyrrolizidine alkaloids (PAs). This botanical was selected by the Genotoxicity Working Group.

In *Comfrey*, PAs include lycopsamine (DTXSID60145542) and 7-acetyllycopsamine (DTXSID50223742) (Mei et al., 2010). Oral comfrey has been banned or restricted in most countries including the US (FDA, 2001), but topical forms i.e. ointments, creams and liniments, (likely depleted for PA content) are available and advertised as useful for wound healing sprains and bone fractures (LiverTox, 2012).

Evidence suggests that at chronic exposure, the active metabolites of PA in comfrey interact with DNA and liver endothelial cells and hepatocytes, resulting In DNA damage, mutation induction, and cancer development (Hirono et al., 1978). PAs have been reported to have toxic developmental and teratogenic reproductive effects. Administration of lasiocarpine (DTXSID1020772), a PA found in comfrey, to lactating rats resulted in liver toxicity and deaths in the suckling pups at doses that had no apparent effects on the dam or their milk production (EFSA, 2011a). However, developmental and reproductive effects of PAs are observed at higher doses than are the carcinogenic effects.

Given the minimal human, animal, or even mechanistic information of comfrey specifically, the developmental and reproductive toxicity has yet to be understood although genotoxic carcinogenicity is recognised to be the driving endpoint for risk assessment of PAs in comfrey.

**Ephedra**

Ephedra, commonly referred to as ma huang, is a genus of gymnosperm shrubs indigenous to China. Traditionally utilized in China and India, it has been employed to alleviate a range of ailments including colds, fevers, and headaches, and is also believed to aid in weight loss. Its biologically active constituents consist of alkaloids such as ephedrine (DTXSID0022985). *Ephedra* species containing ephedrine alkaloids have been linked to multiple potentially severe side effects, including liver injury, and has been banned from sale in the United States and elsewhere (FDA, 2008; LiverTox, 2018). This botanical was selected by the Cardiotoxicity and Neurotoxicity Working Groups.

Few studies have investigated the effects of *Ephedra* species and ephedrine alkaloids on development and reproduction. Ephedrine was given to 3-day chick embryos with or without caffeine and found to have major cardiac malformations. The damage induced by ephedra was potentiated by caffeine (Nishikawa et al., 1985). One study investigated the developmental and reproductive toxicity of other ephedra species, *Ephedra viridis* and *Ephedra nevadensis,* in sheep and cattle (Keeler, 1989). High doses of E. viridis were toxic to pregnant livestock, causing ruminal impaction, diarrhea, vomition, and anorexia, but did not result in congenital deformities in the offspring, indicating no observed developmental toxicity. *E. nevadensis* (tested only in sheep) showed no toxicity and did not affect the offspring either. However, the chemical analysis showed high tannin content in both species but no measurable levels of ephedrine or related alkaloids. EFSA Panel on Food Additives and Nutrient Sources added to Food reviewed ephedra in 2013 (Additives & Food, 2013). They reported that due to an absence of adequate reproductive and developmental studies (and genotoxicity, short-term, and long-term studies) they could not provide advice on the daily intake of ephedra as an herb. They did say that “ephedra herb and its preparations containing ephedra alkaloids used as food supplements were of significant safety concern at the estimated use levels”.

While there is some mechanistic information, without developmental and reproductive studies, it is difficult to assess if *ephedra* is expected to have DART potential.

**Green Tea**

Green tea, made from *Camellia sinensis* leaves, is one of the most common beverages in the world and has numerous studies purporting effects and benefits. Green tea as a beverage is believed to be safe (with natural levels of caffeine) at doses up to eight cups per day. While the tea is considered safe, the BSC focused on concentrated green tea extracts which contain high amounts of catechins. These are commonly used as dietary supplement ingredients. In this project, decaffeinated concentrated green tea extract was selected by the Hepatotoxicity Working Group due to reported adverse event reports.

A study evaluated the maternal and fetal effects of standardized heat-sterilized green tea catechins, which included several catechins including catechin (DTXSID3022322), epicatechin (DTXSID4045133), and epigallocatechin gallate (EGCG, DTXSID1029889) (Morita et al., 2009). These catechins were administered together at doses of 0, 200, 600, and 2000 mg/kg/day to pregnant rats from gestation day 6 to 17. The results showed no test material related deaths or gross abnormalities. However, the highest dose group (2000 mg/kg/day) exhibited reduced body weight gain and feed consumption during early gestation. Despite these maternal effects, administration did not affect uterine weights, intrauterine growth, or survival. No fetal malformations or developmental variations were observed.

Another study in rats evaluated the effects of EGCG, on fetal development and maternal health using high-concentration EGCG extract (Teavigo™) (Isbrucker et al., 2006). Pregnant rats were administered EGCG during organogenesis through diets supplemented with 1400, 4200, or 14,000 ppm. The study found no direct embryo-fetal toxicity, although some maternal toxicity was observed. However, neither dams nor fetuses exhibited toxicity. The results of these studies show that EGCG is non-teratogenic when plasma concentrations reach as high as 191ug/mL. A two-generation study with dietary EGCG preparations of 1200, 3600, or 12,000 ppm showed no adverse effects on reproduction or fertility. However, the highest dose reduced the offspring's growth rate and slightly increased pup loss, while the 3600 ppm dose affected growth in the second generation only. The highest dose tested did delay sexual maturation, likely due to the reduced weight gain in these animals.

An in vitro mouse embryo study evaluated EGCG’s effects on blastocysts, examining attachment, outgrowth, and implantation (Fan & Chan, 2014). EGCG exposure increased apoptosis, reduced total cell number, and lowered implantation success. Post-implantation, EGCG treatment led to higher resorption rates and decreased fetal weight.

A study investigated the effects of green tea and its constituents on zebrafish embryos, reported that green tea increased mortality, delayed gastrulation, and shortened body length. Green tea effected wnt signaling as well (Zhang et al., 2023). Another study assessed the neurodevelopmental effects of the EGCG using human and rat neural progenitor cells in vitro. EGCG was found to disrupt migration distance and pattern, and lead to altered glia alignment and reduced migration of young neurons (Barenys et al., 2017).

Together this is conflicting evidence regarding the effects of concentrated green tea on reproduction and development and while studies would seem to point to slight effects only at high concentrations, more research is needed.

**Kava**

Kava (Piper methysticum) is a plant indigenous to the islands in the South Pacific where it is commonly used to prepare a traditional beverage. Supplements containing the herbal ingredient kava are promoted for relaxation (e.g., to relieve stress, anxiety, and tension), sleeplessness, menopausal symptoms and other uses (Merlin & Lindstrom, 1992; Singh, 1992). Kava was selected based on its available animal studies by the Hepatoxicity Working Group.

The pharmacological properties of kava are postulated to include blockade of voltage-gated sodium ion channels, enhanced ligand binding to gamma-aminobutyric acid (GABA) type A receptors, calcium ion channel blockade, reduced neuronal reuptake of noradrenaline, reversible inhibition of monoamine oxidase B and suppression of eicosanoid thromboxane synthesis ((Pittler & Ernst, 2000; Singh, 1992). Although some clinical trials have concluded Kava extract to be generally well tolerated, spontaneous reports of adverse reactions associated with kava preparations have raised concerns over hepatotoxic concerns (FDA, 2020; Humberston et al., 2003; Stickel & Shouval, 2015). Other reported reactions have included skin reactions, central nervous system effects, and effects on mental performance (Soares et al., 2022).

Kava extracts also show evidence for carcinogenicity in experimental animals, most likely through a non-genotoxic mode of action. Yet, the chemotherapeutic potential of Kava against cancer has also been suggested (Soares et al., 2022). The risk of serious liver toxicity has prompted regulatory actions in the EU, Canada, Australia, and the US to either prohibit or warn of the risks of liver toxicity with kava-containing products. With limited, mechanistic, developmental, and reproductive studies, it is difficult to assess if kava is expected to have DART potential.

**Kratom**

*Mitragyna speciosa* is a tropical tree indigenous to Southeast Asia but is now cultivated elsewhere. In Thailand, the tree and leaf-preparations from it are called kratom. Traditionally, fresh or dried kratom leaves are chewed or made into tea (EUDA, 2024). At a low dose, kratom has stimulant effects, but at higher doses can have sedative-narcotic effects. It has been used in traditional medicine and as an opium substitute. The main psychoactive components in the leaves are two alkaloids: mitragynine (DTXSID701032140) and 7-hydroxymitragynine (DTXSID20903988), which act as selective and full agonists of the μ-subtype opioid receptor. Kratom was selected by the Neurotoxicity Working Group.

Kratom has become frequently used to manage chronic pain, but has the potential to lead to dependence, addiction, and toxicity. Kratom and its constituents can cause a variety of symptoms, including neurologic, metabolic, and psychological symptoms. Adverse events associated with kratom use include hepatotoxicity, seizure and coma, lung injury, and cardiotoxicity. Pregnant patients who use kratom have reported newborns developing neonatal abstinence syndrome (Eggleston et al., 2019; Jentsch & Pippin, 2025). However, few studies have investigated the effects of kratom on developmental and reproductive endpoints.

To evaluate the effects of mitrragynine on adolescent development, male Sprague-Dawley rats were exposed to 3, 10, or 20 mg/kg mitragynine at postnatal day 31 for 15 consecutive days. Mitragynin exposure did not affect recognition memory in a novel object recognition task, but did affect reference memory and social interactions. Metabolomic analysis revealed an effect (Zul Aznal et al., 2022) of kratom on arachidonic acid, pantothenate and CoA, and tryptophan pathways. These findings suggest that adolescent kratom exposure can cause cognitive behavioral deficits that may be associated with changes in the brain metabolite profiles (Zul Aznal et al., 2022).

Zebrafish embryos were exposed to a kratom decoction and its major alkaloids, including mitragynine for 96 hpf. The kratom decoction caused 100% mortality at ≥500 μg/ml and decreased the hatching rate in a concentration-dependent manner. Mytragynine exposure resulted in 100% mortality at 100 μg/ml, and caused significant alterations in the morphological development of zebrafish embryos at lower concentrations (Damodaran et al., 2021). In a study using *C. elegans*, kratom dose-dependently reduced brood size and health of parent worms and their progeny at doses higher than relevant to human consumption. Further, 7-hydroxymitragynine, but not mitragynine, presented with toxic and developmental effects at very high concentrations (Hughes et al., 2022).

Together, these studies suggest that kratom consumption may have potential developmental risks during pregnancy and early life stages and thereby warrants further investigations.

**Yohimbe**

Yohimbe (*Pausinystalia yohimbe*), an evergreen member of the coffee family, originates from the tropical regions of West Africa's coast. Traditionally, its bark has been used to treat fever, leprosy, and coughing. In more recent times, yohimbe has been marketed as both an aphrodisiac and a supplement to enhance athletic performance. Its biologically significant components include indole alkaloids like yohimbine (DTXSID9040130). Yohimbe was selected for its expected neurotoxicity effects, including being associated with heart attack and seizures (NCCIH, 2020).

In a study of adult male mice, yohimbe bark suspension was administered varying doses (188, 375, or 750 mg/kg-day) for 90 days (Al-Majed et al. 2006). Post-treatment, males were mated with untreated females, and assessments included reproductive organ weight, sperm characteristics, pregnancy rates, and testes cytology. At the highest dose, mice exhibited an increase in seminal vesicle weight, reduced sperm motility and count, higher abnormal spermatozoa, and elevated levels of estradiol, prolactin, and testosterone.

Despite these observations suggesting potential reproductive dysfunction, the EFSA Panel on Food Additives and Nutrient Sources Added to Food Scientific Opinion on yohimbine highlights study's inconsistent findings, especially in dominant lethal tests showing varied post-implantation losses, prevent drawing definitive conclusions about yohimbe's effects on male fertility (Chain, 2013). The National Center for Complementary and Integrative Health (NCCIH) says yohimbe may be “unsafe to use yohimbe orally … during pregnancy and while breastfeeding.” However, there is no referenced evidence to back this up, so it is likely a cautionary suggestion in the absence of data.

The 2013 EFSA panel concluded that yohimbe is missing a lot of toxicity data, and for its key constituents, including for subchronic, genotoxicity, and reproductive toxicity.

In the decade since there have been some studies on yohimbe and yohimbine, yet there is still not enough information to predict if yohimbe will have developmental or reproductive effects.
